# Genome-wide detection of superior haplotypes for seed oil and protein content in Northeast China soybean (*Glycine max* L.) germplasm

**DOI:** 10.3389/fpls.2026.1767299

**Published:** 2026-02-24

**Authors:** Moran Bu, Ye Zhang, Weitao Xu, Yanhua Li, Hui Yu, Yaohua Zhang, Suxin Yang, Javaid Akhter Bhat, Xianzhong Feng

**Affiliations:** 1 Key Laboratory of Soybean Molecular Design Breeding, Northeast Institute of Geography and Agroecology, Chinese Academy of Sciences, Changchun, Jilin, China; 2 College of Advanced Agricultural Sciences, University of Chinese Academy of Sciences, Beijing, China

**Keywords:** GWAS, haplotype, Northeast China, oil content, protein content, soybean

## Abstract

Seed oil content (SOC) and seed protein content (SPC) are the crucial traits determining the economic importance of soybeans. However, the molecular mechanism underlying the high SOC and low SPC of Northeast China soybeans is still limited. To address this, we elucidated the genetic basis of SOC and SPC in soybean germplasm adapted to Northeast China by employing an integrated genomic analysis. The genome-wide association study (GWAS) detected 105 and 59 significant SNPs associated with the SOC and SPC, respectively across four environments plus combined environment (CE). The haplotype allele number in the 15 identified haplotype blocks varied from 2–4 regulating the SOC and SPC in the range of 16.68-21.15% and 38.63-42.69%, respectively. Five quantitative trait loci (QTLs) among the total 17 identified QTLs were novel that include *qSOC1*, *qSPC1*, *qSOC9*, *qSOC_SPC15.1* and *qSOC_SPC15.2* associated with SOC or/and SPC. Based on the *in-silico*, variant annotation and haplotype analysis, the 80 genes were prioritized as potential candidates. The haplotype alleles of these genes varied from 2–8 regulating SOC and SPC in the range of 15.98-21.23% and 37.69%-43.30%, respectively. Twelve of 80 genes showed distinct selection signatures between the two populations, suggesting their key roles in shaping the specific seed quality profiles of soybean germplasm in Northeast China. Hence, the current study provides novel insights on divergent breeding influencing the local adaptation and seed quality difference between different regional soybean populations. Besides, the stable QTLs, superior haplotypes and candidate genes identified can be used for soybean improvement.

## 1 Introduction

Soybean possesses high nutritional significance as their seeds have high content of oil and protein; and the average seed oil content (SOC) and seed protein content (SPC) in soybean is 20% and 40%, respectively (Guo et al., 2022). These features make the soybean as the fifth major crop in the world production, and is cultivated globally (Karges et al., 2022). Soybean oil is consumed by humans as well as used for industrial applications; besides, soybeans provide plant proteins to humans and livestock’s; hence, the improvement of the SOC and SPC has central importance in the soybean breeding program (Majidian et al., 2024). Although, conventional breeding has made considerable efforts in the development of soybean cultivars with high SOC, these achievements have been made at the cost of reducing the SPC and vice versa (Li et al., 2019). The significant strong negative correlation between SOC and SPC has made it difficult for the conventional breeding to simultaneously improve these both traits in the same soybean cultivar (Mo et al., 2024). In this context, the molecular breeding can allow the simultaneous improvement of SOC and SPC in the same cultivar by using the independent or non-correlated MTAs (marker-trait associations)/QTLs (quantitative trait loci)/genes (Nair and Rabbani, 2024). However, molecular breeding requires the elucidation of detailed genetic architecture for these traits. Identifying and confirming the independent genetic loci associated with the SOC and SPC will allow the development of soybean cultivars with high seed oil and protein content; that in turn will increase the economic value and consumer acceptance of the soybeans.

Although, substantial attempts have been made in the past to detect the genetic elements associated with SOC and SPC traits in soybean; to this end, ~330 and 250 QTLs linked with SOC and SPC, respectively are deposited in the SoyBase (http://www.soybase.org). Mostly the classical approach of linkage mapping involving the use of biparental population which possesses the lower number of recent recombination events has been utilized for the detection of these QTLs, thus has the limitation of low-resolution and larger confidence interval (Capilla-Pérez et al., 2024). The disadvantage of low-resolution has in turn restricted the use of these QTLs in the practical soybean breeding for the enhancement of SOC and SPC (Karikari et al., 2019). In this context, the association mapping approach has the benefits of higher resolution and precision because it uses the diverse natural population that has undergone recombination over thousands to millions of years, thus has emerged as a more realistic method for molecular breeding applications (Leventhal et al., 2025). There are multiple studies in which the association mapping/GWAS (genome-wide association study) has been used for the SOC and SPC traits in soybean; and majority of these studies were performed in low-latitude regions. For example, Li et al. (2019) performed the association mapping for SOC and SPC traits by using 185 diverse accessions of soybean that were genotyping with SLAF-seq technology; and they identified 31 MTAs linked with SOC and SPC distributed on the 12 out of total 20 soybean chromosomes. Similarly, Lee et al. (2019) used the 621 diverse accessions of soybean in the GWAS analysis of SOC and SPC, and this panel was genotyping using SoySNP50K array; and they detected 19 and 16 significant MTAs linked with the SOC and SPC, respectively. Chen et al. (2025) used the nested-association population (NAM) of 1107 soybean accessions genotyping with SoySNP50K array, and they detected 104 and 99 MTAs associated with SOC and SPC, respectively. Moreover, Doszhanova et al. (2024) used 252 diverse soybean accessions genotyping with whole genome resequencing (WGRS), and they identified nine and nine MTAs linked with the SOC and SPC, respectively. There are some other studies that have used the GWAS for SOC and SPC traits in low-latitude regions (He et al., 2021; Kim et al., 2023; Yuan et al., 2024; Patel et al., 2025). However, some studies of GWAS have been carried out for SOC and SPC for soybeans in high-latitude regions. For instance, Zhang et al. (2025) have used the four-way recombinant inbred line (FW-RIL) populations for GWAS of SPC, and they have identified 18 MTAs linked with SPC.

The SOC and SPC being the complex traits are regulated by numerous genes, and limited attempts have been made to identify the actual genes governing the SOC and SPC in soybean. Hence, there is a need to detect actual genes governing these traits in soybean. Genes such as *GmSWEET10*, *GmOLEO1* and *GmST05/GmMFT* regulate the SOC positively, but they regulate the SPC negatively (Zhang et al., 2019; Wang et al., 2020; Mukherjee et al., 2023). In contrast, the genes such as *POWR1-TE*, *GmJAZ3* and *GmSDP1* positively regulate the SPC but negatively regulate SOC (as reviewed by Mo et al. (2024)). The *GmSop20* has been reported to serve as a key gene regulating the SOC/SPC ratio in soybean (Zheng et al., 2025). Moreover, the *ST1* gene has been documented to regulate SOC in soybean (Li et al., 2022). The biosynthesis of fatty acids and amino acids are significantly determined by the allocation of the nitrogen and carbon sources, and breakdown of carbohydrate substances are the major source for providing the materials in the synthesis of oil and protein. The *GmSWEET10* (*Glyma.15G049200*) is a sugar transporter gene that controls the SOC and SPC accumulation (Wang et al., 2020). Besides, the SPC of soybean is regulated by other proteins such as GTPase (encoded by *GmRab5a*), guanyl-nucleotide releasing factors and methionine lyase (Wei et al., 2019). Hence, substantial attempts are required to detect the genes regulating SOC and SPC in soybean.

In the past, association mapping has predominantly utilized the biallelic single nucleotide polymorphism (SNP) markers, which has greatly decreased the efficiency of GWAS because it can capture only two alleles in the diverse soybean germplasm. But there are multiple alleles of the genetic loci or gene across the diverse soybean panel, which might be rare and superior, and go uncaptured (Liu et al., 2025). In this context multiallelic haplotype markers can overcome these challenges; and the better performance of haplotype markers relative to the SNP markers are documented through both empirical and simulation methods (Yu et al., 2024). The superior and rare haplotypes are reported for various agronomic traits in rice (Abbai et al., 2019) and stress tolerance in rice and pigeonpea (Kuroha et al., 2018; Sinha et al., 2020). In soybean, the superior haplotypes are detected for plant height, yield-related traits and branch number (Liang et al., 2022; Shao et al., 2022; Qin et al., 2023). However, almost no efforts have been made in the detection of superior haplotypes for SOC and SPC traits in soybean. Hence, there is a need to identify these genetic elements for these traits in soybean.

It has been documented that SOC and SPC exhibit a significant latitudinal gradient (Guo et al., 2022). The SOC has direct relationship with the latitude i.e., SOC increases with the latitude in soybean; in contrast, SPC is negatively correlated with latitude (Rotundo et al., 2016). For example, it has been documented that SOC and SPC are low and high, respectively, in the soybeans grown in the Southern US relative to those cultivated in Northern US (Rotundo et al., 2016). In China, the SOC and SPC are high and low, respectively in the soybeans cultivated in Northeast China compared to those grown in Huanghuaihai region and southern parts of China (Song et al., 2023). The varied environmental conditions across the different latitudes significantly affect the SOC and SPC in soybeans. The cool weather and long-day conditions are favorable for oil biosynthesis, while warm weather and short-day conditions are favorable for protein accumulation (Kalantar Ahmadi and Sarhangi, 2025). In addition, delayed sowing may result in a decrease of SPC, which is the important reason in the high-latitude regions (Zelaya Arce et al., 2025). In total, the environmental factors in high-latitude regions such as lower temperature, reduced growth season and environmental stress factors enforce the soybean plants to biosynthesis and accumulate more SOC relative to SPC as a strategy for adaptation. This can be explained as, in the high-latitude regions of Northeast China, the temperature is low at the sowing time and maturity time; hence, presence of the high SOC rich in unsaturated fatty acids can allow the normal germination of the soybean seeds as well as the survival of the seeds at maturity time. Because the oil can be in liquid form and not frozen at the low temperature, thus will be the most suitable source of energy in the high-latitudes during the seed germination (Zhou et al., 2025). Hence, the soybeans adapted to Northeast China/high-latitudes have genetic adaptations that favor oil production, as this could improve the plant’s ability to endure harsh climates. This could come at the cost of protein content, which may be less of a priority for the plant in extreme conditions.

Here, we utilized a diverse collection of soybean accessions adapted to the Northeast region of China to investigate the genetic basis of SOC and SPC. To achieve this, we employed a combined strategy that integrated association mapping, haplotype analysis, QTL analysis and candidate gene analysis. The results of our investigation revealed significant SNPs, superior haplotypes, stable genomic loci/QTLs and candidate genes associated with SOC and SPC in soybean. The detected SNPs, superior haplotypes, genomic loci and genes could be potentially deployed in soybean breeding programs aimed at producing soybean cultivars with both high SOC and SPC. Besides, our results revealed divergent breeding of key genes may result in local adaptation and the specific quality profile of soybeans in Northeast China.

## 2 Materials and methods

### 2.1 Plant materials and field experiments

Here, a set of 340 diverse accessions of soybean derived from the different growing regions of China were used as plant material (**Supplementary Table S1**). These soybean accessions were planted under natural conditions at two separate locations of the Jilin province viz., Changchun (CC) and Jiutai (JT). In the CC location, the accessions were grown in the experimental field of the Northeast Institute of Geography and Agroecology, Chinese Academy of Sciences (44°0’N, 125°24’E), on the following dates: April 29^th^, 2022 (CC22); April 28^th^, 2023 (CC23); and April 30^th^, 2024 (CC24), respectively; whereas these accessions were grown on May 10^th^, 2024 in the experimental field of Changchun Polytechnic (44°4’N, 125°40’E, Jiutai District, Changchun), representing the environment JT24. Hence, the diverse soybean germplasm resources were accessed across the four different environments viz., CC22, CC23, CC24 & JT24. The randomized complete block design (RCBD) involving three replications was used for planting this germplasm. The plot of each soybean accession was consisted of three rows with the spacing between the rows maintained at 50 cm and the length of row kept at 200 cm. At the maturity stage, the seeds of each accession were harvested in the main season of all four environments. The soybean germplasm was cultivated following the normal agronomic practices.

### 2.2 Phenotypic analysis of SOC and SPC

The seed samples collected were placed in a cup with a bottom of 5 cm in diameter, and a height of 3 cm for near-infrared spectroscopy analysis; and the seeds were analyzed without any treatment and in good condition. The cup was filled with the equivalent of about 60 ml of soybean seed (about 20 g or 80 seeds). Spectra acquisition was carried out using a Fourier transform near-infrared (FTNIR) analyzer (Antaris II spectrometer: Thermofisher Scientific, France). The SOC and SPC were expressed in percentage by weight of seeds. Phenotypic data of SOC and SPC were estimated for each accession in three replications for all four environments. The SOC and SPC were estimated in each replication, and the average of three replications was used for further analysis. The combined environment (CE) data were derived from the data of individual environments by utilizing the “lme4” package in R environment (Bates et al., 2015). The predicted means (best linear unbiased predictions, BLUPs) for the CE were calculated by fitting the following model:


$$Y_{ijk}=\;\mu +G_{i}+E_{j}+GE_{ij}+Rep_{k}\left(E_{j}\right)+\epsilon_{ijk}$$

where $Y_{ijk}$ is the phenotypic value of the *i*-th genotype in the *k*-th replication of the *j*-th environment, μ is the overall mean, $G_{i}$ is the random effect of the *i*-th genotype, $E_{j}$ is the random effect of the *j*-th environment, $GE_{ij}$ is the random effect of the genotype-by-environment interaction, $Rep_{k}\left(E_{j}\right)$ is the random effect of the block nested within the *j*-th environment, and $\epsilon_{ijk}$ is the random error. The analysis of variance (ANOVA) was calculated by utilizing the R language. The broad-sense heritability (*H*) was estimated as $\sigma_{G}^{2}/\left(\sigma_{G}^{2}+\sigma_{GE}^{2}/n+\sigma_{\epsilon }^{2}/nr\right)$, in which the $\sigma_{G}^{2}$, $\sigma_{GE}^{2}$ and $\sigma_{\epsilon }^{2}$ represent the genotypic variance, genotype × environment interaction variance, and residual variance, respectively, *n* is the number of environments, and *r* is the number of replications within each environment.

### 2.3 Genotyping of plant materials

The resequencing data for the soybean accessions were obtained from our previously published study (Zhang et al., 2024b). Briefly, the genomic DNA was extracted from fresh and healthy leaf tissues of three-week-old seedlings using the cetyltrimethylammonium bromide (CTAB) method (Murray and Thompson, 1980). The genomic DNA was fragmented to a target size of 350 bp. Library preparation was performed by Gene Denovo Biotechnology Co., Ltd. (Guangzhou, China) sequenced on the DNBSEQ-T7 platform (BGI, Shenzhen, China) by Gene Denovo Biotechnology Co., Ltd. (Guangzhou, China). The average depth of resequencing was 25 × and the volume of the total data produced was 8.57 Tb. The fastp software (Chen et al., 2018) was utilized to eliminate the reads with low quality, reads with > 10% N content and adapter sequences; and we retained only the clean reads. The Burrows-Wheeler Aligner software (Li, 2013) was utilized to map the sequence reads with the reference genome of Wm82.a2.v1. The Genome Analysis Toolkit (McKenna et al., 2010) was used to identify the sequence variants such as SNPs, insertions/deletions (InDels) and other larger variants; and in this way we identified a total of 25,442,248 genetic variants. Furthermore, we utilized the stringent measures of quality control viz., missing rate greater than 10% and minor allele frequency less than 0.05, and finally detected high-quality SNPs of 3,343,372, with the mean heterozygous rate of 0.094.

### 2.4 Population structure and linkage disequilibrium analysis

The PCA result and kinship of GWAS panel were generated by GAPIT package (Wang and Zhang, 2021) in R environment. The PopLDdecay software v3.43 (Zhang et al., 2018a) was used for LD decay calculation with the following parameters: -MaxDist 300, -MAF 0.05, and -Miss 0.1.

### 2.5 Association mapping analysis

For association mapping analysis, the study utilized the GAPIT package (Wang and Zhang, 2021). Seven different GWAS models were used viz., GLM (General Linear Model), MLM (Mixed Linear Model), CMLM (Compressed Mixed Linear Model), MLMM (Multi-Locus Mixed Linear Model), SUPER (Settlement of MLM Under Progressively Exclusive Relationship), FarmCPU (Fixed and random model Circulating Probability Unification) and BLINK (Bayesian-information and Linkage-disequilibrium Iteratively Nested Keyway). The multiple models were used because various models of GWAS are built on divergent hypotheses involving different distribution characteristics of QTL effects. Among these, MLMM, BLINK, SUPER, and FarmCPU are considered multi-locus models, whereas GLM, MLM and CMLM are categorized as single-locus GWAS models. The default parameters of GAPIT were employed to detect significant SNP associations. The GAPIT package in the R program was employed for conducting principal component analysis (PCA) and kinship analysis to assess population structure (Lipka et al., 2012). To visualize population structure in R environment, we used the “plotly” package. A suggestive association threshold (i.e., 1/*N_test_*) was set at *P* < 2.99 × 10^-7^, which reflects the level at which, under the null hypothesis, one false positive result is expected across the entire genome scan (Lander and Kruglyak, 1995; Duggal et al., 2008; Wang et al., 2012; Li et al., 2013; Guo et al., 2017). The UpSetR package was used to draw the UpSet plots (Conway et al., 2017).

### 2.6 Haplotype analysis

To determine the level of LD between pairs of SNPs, we employed the Haploview 4.2 software (Barrett et al., 2004). A haplotype block was determined when two or more than 2 significant SNPs were present in the same chromosome, and these SNPs were present within the LD decay distance in the upstream and downstream of chromosome such as ± 71.7 kb in the present study. Pairwise means were analyzed using Fisher’s Least Significant Difference (LSD) Test, and visualization of the results was performed in the R environment.

### 2.7 Candidate gene identification

Gene models that were present across the genomic interval of 17 QTLs along with their function annotation were collected from SoyBase (https://www.soybase.org/); and soybean genome version of Wm82.a2.v1 was utilized to collect the gene model information. The information about gene ontology (GO) was obtained from SoyBase (https://www.soybase.org/). The analysis of GO enrichment was carried out by utilizing the agriGO V2.0 (https://systemsbiology.cau.edu.cn/agriGOv2/) tool (Tian et al., 2017). The publicly available RNA-seq data at SoyMD (https://yanglab.hzau.edu.cn/SoyMD/#/) for different tissues/organs as well as development stages were collected for the model genes (Yang et al., 2023). The heatmap of transcriptomic data was produced by utilizing the ComplexHeatmap package of R software (Gu et al., 2016). Initial screening of the putative candidate genes was carried out using the *in-silico* analysis and a literature survey. The genes selected based on the initial step were further screened using the variant annotation and haplotype analysis; and the genes fulfilling the criteria of variant annotation and haplotype differences were prioritized as potential candidate genes regulating the SOC and SPC in soybean. The variant annotation was carried out by utilizing the SnpEff software (Cingolani et al., 2012).

The sequencing data for the haplotype selection analysis of soybean accessions adapted to different latitudes of China has been collected from SoyMD (https://yanglab.hzau.edu.cn/SoyMD/#/). The different regions of China have been divided into China-I, China-II, China-III, China-IV, China-V and China-VI (Liu et al., 2023); China-I has the highest latitude including the Northeast China; China-II has the mid-latitude that includes Huanghuaihai regions; and China-III to China-VI include the Southern China and have low-latitude. Pairwise *F_ST_* values were estimated by using the window size of 5 kb and a step size of 200 bp using VCFtools (Danecek et al., 2011). Nucleotide diversity (*π*) values within various populations were also evaluated using VCFtools (Danecek et al., 2011) with a fixed window of 5 kb and a step size of 200 bp. Tajima’s *D* values were evaluated using VCF-kit (Cook and Andersen, 2017) with a fixed window of 5 kb and a step size of 200 bp.

## 3 Results

### 3.1 Phenotypic analysis of SOC and SPC

**Figure 1:**
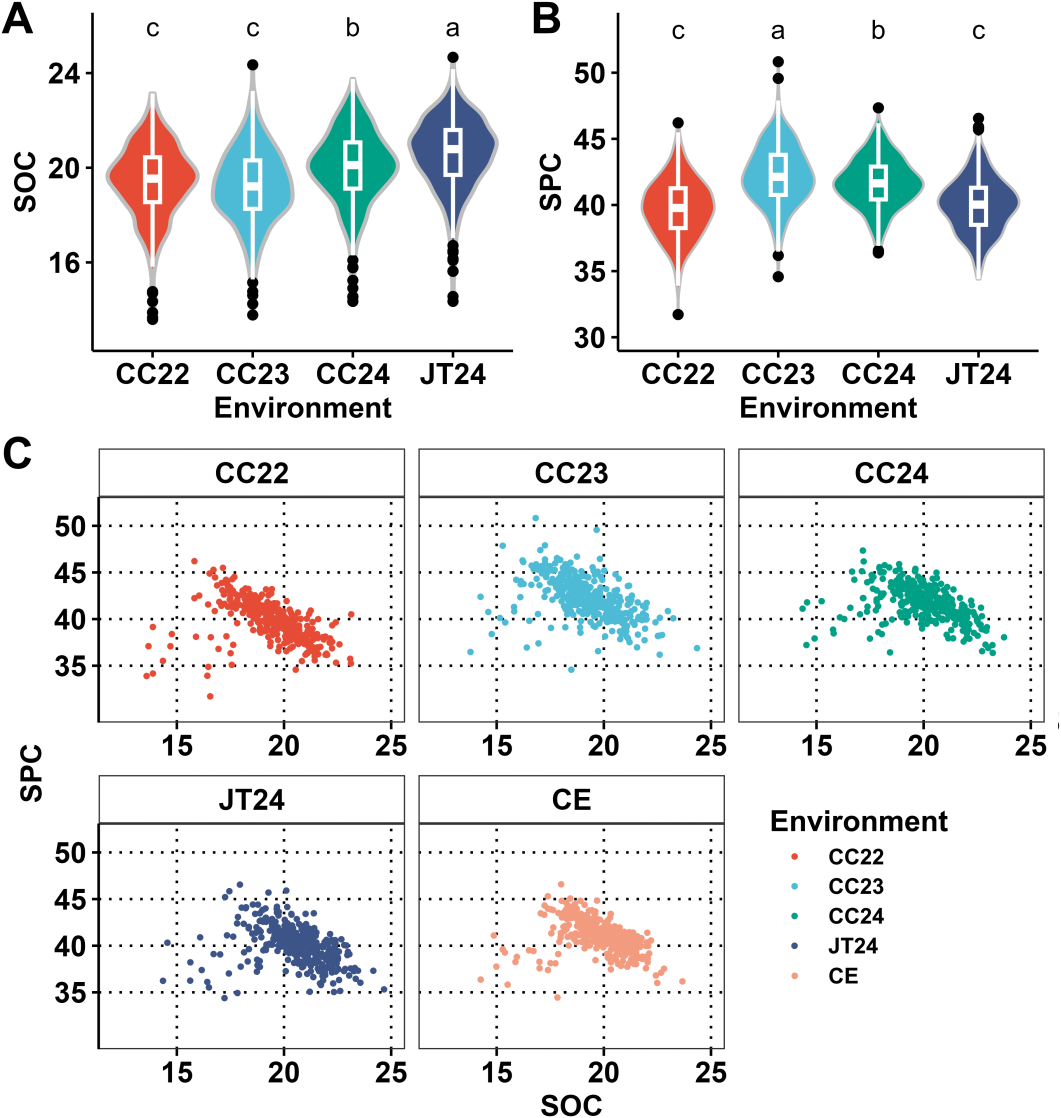
Comparison of trait values and correlation analysis of SOC and SPC across 340 soybean accessions. **(A)** Comparison of average values of the SOC across four individual environments viz., CC22, CC23, CC24 & JT24 in the soybean germplasm. **(B)** Comparison of average values of the SPC across four individual environments viz., CC22, CC23, CC24 & JT24 in the soybean germplasm. **(C)** Scatter plot of SOC and SPC traits in the soybean germplasm across the four individual environments (CC22, CC23, CC24 & JT24) plus combined environments (CE). The different letters at the top of density curve correspond to differences at the significant level, whereas the same letters correspond to differences at the non-significant levels.

The mean values of the SOC and SPC across the four different environments, viz. CC22, CC23, CC24 and JT24 are revealed in the violin plots (**Figures 1A, B**; **Supplementary Figure 1**). The average values of SOC showed significant differences among the four environments; however, the average values of SOC exhibited non-significant differences between CC22 and CC23, while the remaining comparison groups of environments showed significant differences in the average values of SOC (**Figure 1A**). The average values of SPC revealed significant differences among the four environments, but the non-significant differences were observed between CC22 and JT24 for the SPC; while the significant differences were observed for the average values of SPC in the remaining comparison groups (**Figure 1B**). The frequency distributions of SOC and SPC traits in the set of 340 diverse soybean accessions across four individual environments, viz. CC22, CC23, CC24 & JT24, are shown in **Supplementary Figure 1**. The scatter plots of SOC and SPC traits in CC22, CC23, CC24, JT24 environments and the CE shown in **Figure 1C** and the Pearson correlation coefficients between these traits in different individual environments and the CE (-0.41, -0.46, -0.41, -0.36 and -0.34, *P* < 0.0001) underscored a consistent moderate negative correlation between SOC and SPC across different environments in our soybean population.

The parameters of descriptive statistics for the SOC and SPC traits in 340 soybean cultivars across four individual environments, viz. CC22, CC23, CC24 & JT24 plus the CE, are presented in **Supplementary Table S2**. The average values of the SOC ranged from 19.21 to 20.56% in the environments of CC23 and JT24, respectively; and the minimum and maximum values for the SOC were 13.59% and 24.67% in the environments of CC22 and JT24, respectively. The coefficients of variation (CVs) of SOC varied from 7.62 to 8.37% in the environments of JT24 and CC23, respectively; and the CV of SOC in the CE was 7.11%. The skewness of the SOC varied from -0.79 to -0.21 in the JT24 and CC23 environments, respectively; and the kurtosis of SOC ranged from 0.52 to 1.37 in the environments of CC23 and CE, respectively. Broad-sense heritability (*H*) for SOC was high equal to 0.86 (**Supplementary Table S2**). The results of ANOVA analysis showed highly significant (*P<0.0001*) variances of genotype (*G*), environment (*E*) and genotype × environment (*G × E*) interaction for the SOC (**Table 1**).

**Table 1:** Analysis of variance (ANOVA) for SOC and SPC in combined environment.

Trait	Source	DF	SS	MS	*F* value	*P* value (Prob > *F*)
SOC	Block within *E*	10	24.406	2.441	4.543	<0.0001 ***
	Genotype(*G*)	339	8975.567	26.476	49.286	<0.0001 ***
	Environment(*E*)	3	1161.514	387.171	720.719	<0.0001 ***
	*G* × *E*	1017	2413.120	2.373	4.417	<0.0001 ***
	Residuals	3201	1719.582	0.537		
SPC	Block within *E*	10	51.188	5.119	3.763	<0.0001 ***
	Genotype(*G*)	339	14372.447	42.397	31.165	<0.0001 ***
	Environment(*E*)	3	5126.621	1708.874	1256.154	<0.0001 ***
	*G* × *E*	1017	5845.622	5.748	4.225	<0.0001 ***
	Residuals	3201	4354.645	1.360		

DF, degrees of freedom; SS, sum of squares; MS, mean sum of squares; E, environment; Prob, probability; *P* value< 0.0001, significant.

The average values of the SPC ranged from 39.75 to 42.23% in the environments of CC22 and CC23, respectively; and the minimum and maximum values of SPC were 31.72% and 50.83% in the environments of CC22 and CC23, respectively. The CVs for the SPC varied from 4.58 to 5.59% in the environments of CC24 and CC22, respectively; and the CV of SPC in the CE was 4.36%. The skewness for the SPC ranged from -0.11 to 0.02 in the CC22 and CC23 environments, respectively; and the kurtosis of SPC varied from 0.02 to 0.50 in the environments of CC24 and CC23, respectively. Broad-sense heritability (*H*) for SPC was high, and was equal to 0.79 (**Supplementary Table S2**). The results of ANOVA analysis showed highly significant (*P<*0.0001) variances of *G*, *E* and *G × E* interaction for the SPC (**Table 1**).

### 3.2 Population structure and linkage disequilibrium analysis

**Figure 2:**
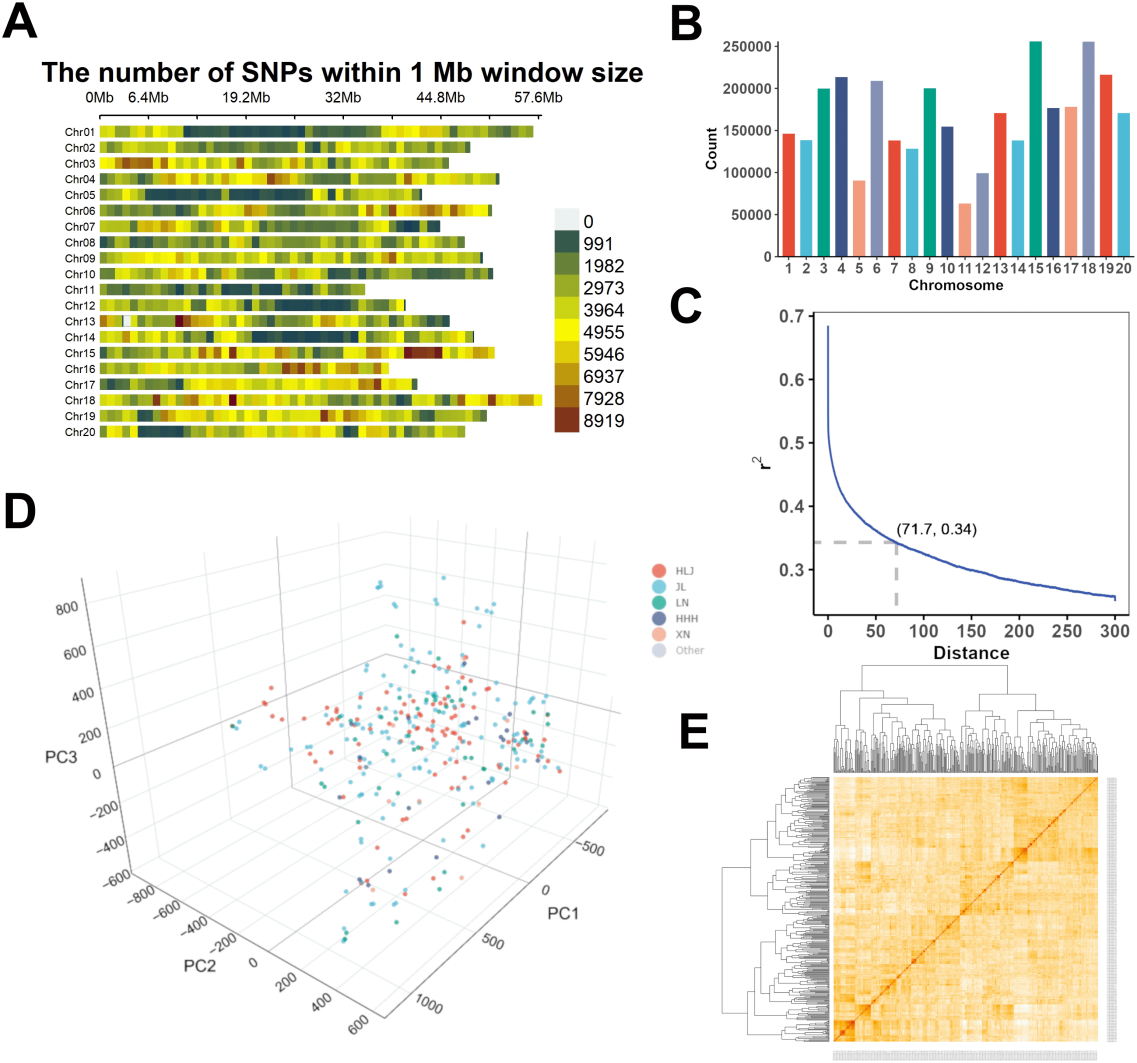
Marker allocation, population structure analysis and LD decay plot in the 340 soybean accessions. **(A)** SNP density plot of 20 soybean chromosomes involving 3,343,372 SNPs across the GWAS panel. Horizontal axis shows the length of chromosomes in Mb, and vertical axis represents the chromosome number; and the various colors depict the chromosome density. **(B)** Distribution of 3,343,372 SNPs across the 20 different chromosomes of soybean. **(C)** LD decay plot generated from the 3,343,372 SNPs in the GWAS panel. Blue thick line corresponds to LD decay fitted with a smoothing spline regression model. The intersection of the horizontal and vertical dotted lines on the LD decay curve represents the LD half decay (*r^2^* = 0.34) and the genetic distance at half decay point is 71.7 kb. **(D)** PCA analysis displays the organization of the 340 accessions of soybean derived from different regions of northeast China. **(E)** Kinship plot displays the relationship among the panel of 340 soybean accessions.

Here, we detected 3,343,372 high-quality SNPs by applying the stringent measures of quality control (**Supplementary Table S3**). The distribution of these SNPs was located across the whole genome of the soybean; the lowest and highest SNPs were present on Chr.11 (63,224) and Chr.15 (256,000), respectively, with an average number of 167,168.6 SNPs per chromosome (**Figure 2B**; **Supplementary Table S3**). Substantial variation was observed for the marker density among the various soybean chromosomes; for example, the lowest marker density of 1,818.51 SNPs/Mb was observed on Chr.11, and the highest marker density of 4,946.25 SNPs/Mb was observed on Chr.15, and the mean value of the marker density was 3,522.37 SNPs/Mb (**Figure 2A**; **Supplementary Table S3**). The kinship matrix showed no clear-cut clustering in the panel of soybean accessions (**Figure 2E**). Besides, no distinct structure was observed, and a continuous distribution of the soybean accessions has been shown by the analysis of population structure (**Figure 2D**; **Supplementary Table S3**).

We studied the features of LD across the panel of 340 diverse soybean accessions, and the outcomes are revealed in the graph (**Figure 2C**). The average value of *r^2^* was 0.32, with LD decay started at 0.68; the half decay reached at 0.34. The intersection occurs between the LD decay curve and half decay at the 71.7 kb; and this distance decides the linkage at the genome-wide level. The genomic region of ± 71.7 kb across the stable significant SNPs was designated as the QTL interval.

### 3.3 GWAS analysis of SOC and SPC

**Figure 3:**
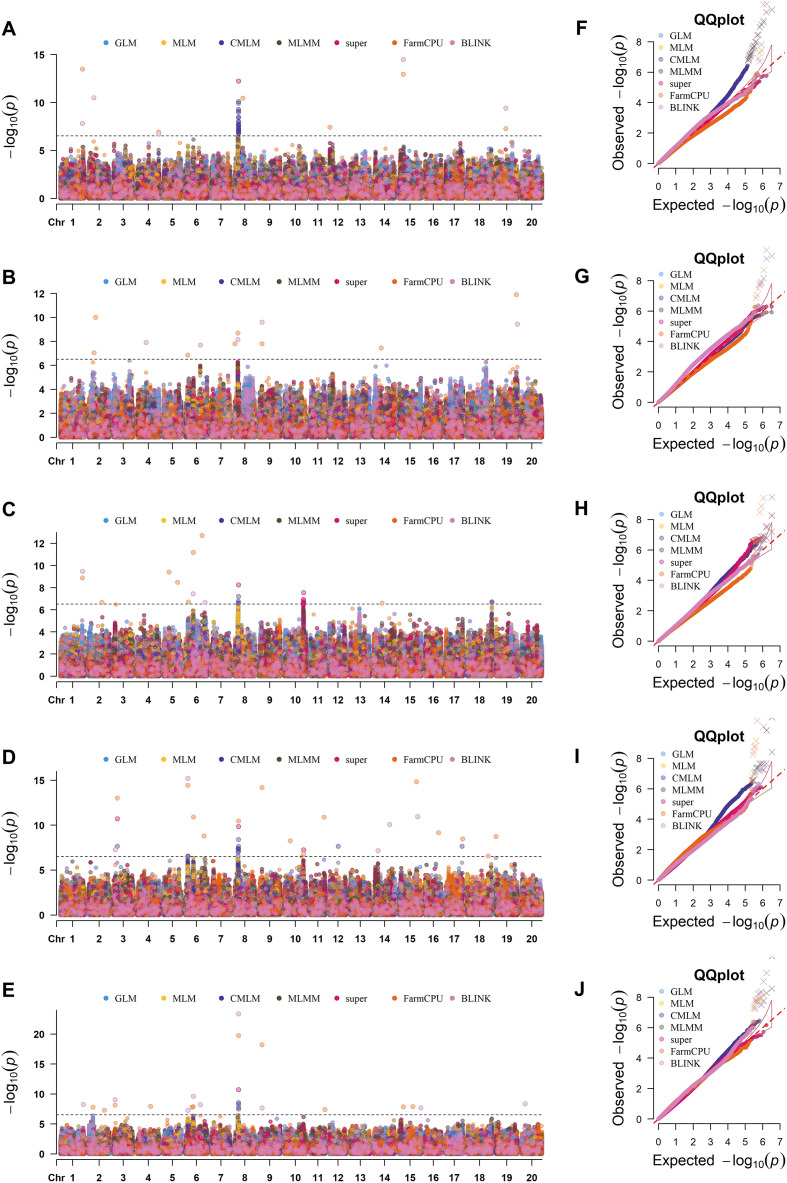
Illustration reveals the GWAS signals of SOC across various GWAS models and environments. The different models include GLM, MLM, CMLM, MLMM, BLINK, SUPER and FarmCPU; and different single environments include CC22, CC23, CC24 & JT24 plus CE. **(A–E)** represents the Manhattan plots in various environments viz., **(A)** CC22, **(B)** CC23, **(C)** CC24, **(D)** JT24 and **(E)** CE. **(F–J)** represents the quantile–quantile (Q–Q) plots in various environments viz., **(F)** CC22, **(G)** CC23, **(H)** CC24, **(I)** JT24 and **(J)** CE. Significant threshold level [-log_10_*P*> 6.52] is shown by the black horizontal dotted line. The number along the X-axis corresponds to different chromosomes. In the Manhattan plot and Q-Q plot different colors of dots correspond to various GWAS models.

**Figure 4:**
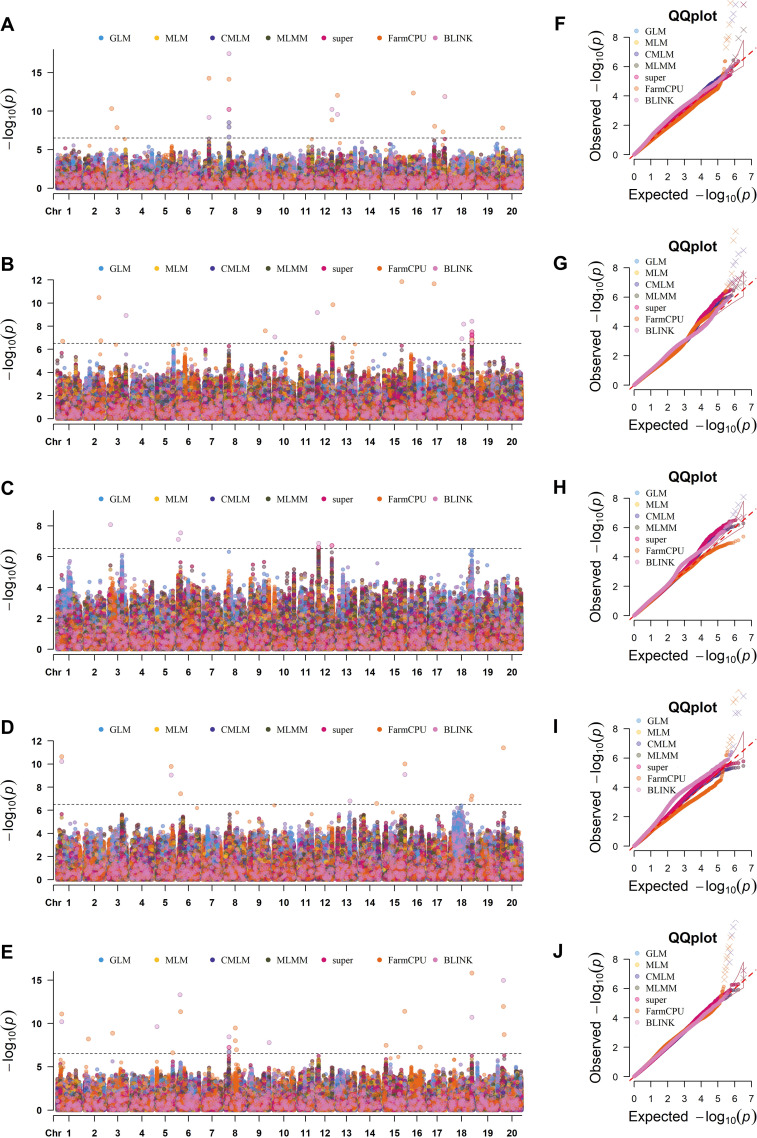
Illustration is revealing the GWAS signals of SPC across various GWAS models and environments. The different models include GLM, MLM, CMLM, MLMM, BLINK, SUPER and FarmCPU; and different single environments include CC22, CC23, CC24 & JT24 plus CE. **(A-E)** represents the Manhattan plots in various environments viz., **(A)** CC22, **(B)** CC23, **(C)** CC24, **(D)** JT24 and **(E)** CE. **(F–J)** represents the quantile–quantile (Q–Q) plots in various environments viz., **(F)** CC22, **(G)** CC23, **(H)** CC24, **(I)** JT24 and **(J)** CE. Significant threshold level [-log_10_*P*> 6.67] is shown by the black horizontal dotted line. The number along the X-axis corresponds to different chromosomes. In the Manhattan plot and Q-Q plot different colors of dots correspond to various GWAS models.

**Figure 5:**
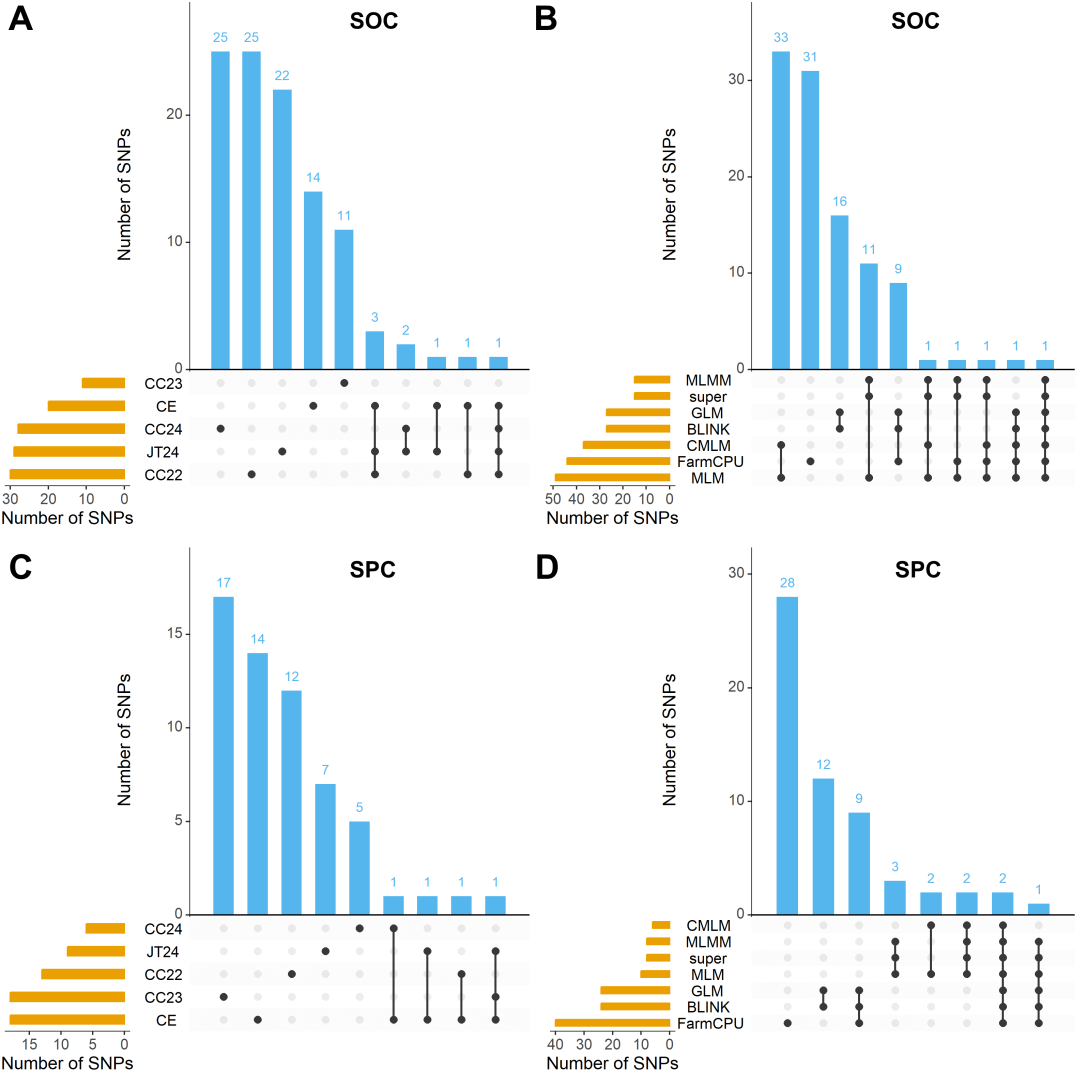
UpSet plots depicting intersection of significant SNPs of SOC and SPC across models and environments. The GWAS models include GLM, MLM, CMLM, MLMM, SUPER, FarmCPU and BLINK; and environments include CC22, CC23, CC24, JT24 & CE. **(A)** UpSet plot demonstrating the intersection of significant SNPs of SOC across various environments. **(B)** UpSet plot demonstrating the intersection of significant SNPs of SOC across various GWAS models. **(C)** UpSet plot demonstrating the intersection of significant SNPs of SPC across various environments. **(D)** UpSet plot demonstrating the intersection of significant SNPs of SPC across various GWAS models.

Here, we detected 105 and 59 significant SNPs linked with the SOC and SPC traits, respectively across the four individual environments (CC22, CC23, CC24 & JT24) plus CE and seven GWAS models (**Figures 3**, **4**; **Supplementary Table S4**). The 105 significant SNPs of SOC were distributed across all the soybean chromosomes except Chr.07 and Chr.15; and the allocation of these SNPs in the various models and environments were presented in UpSet plot (**Figures 5A, B**). A maximum of 33 significant SNPs of SOC were present on Chr.08, and a minimum of one significant SNP was identified on Chr.16 and Chr. 20. The remaining 15 chromosomes possessed significant SNPs in the range of 2 to 15 (**Supplementary Table S4**). By considering the detection of SOC related significant SNPs by various GWAS models, the one SNP viz., Chr08_9071263 was persistently identified by all the seven models viz., GLM, MLM, CMLM, MLMM, SUPER, FarmCPU and BLINK; the rest of the 104 SOC related significant SNPs were identified by one to five GWAS models (**Supplementary Table S4**). Furthermore, the SNP Chr08_9071263 was persistently identified by three single environments plus CE. The SNPs viz., Chr08_9054741, Chr08_9056324 and Chr08_9071236 were persistently detected in two single environments plus CE; and Chr09_6929070, Chr10_44584365, Chr10_44584422 and Chr15_10147967 were detected by two single environments or one single environment plus CE. The rest of the 97 SOC related significant SNPs were identified in one single environment or CE (**Supplementary Table S4**).

The 59 significant SNPs detected for the SPC were distributed across the 17 out of total 20 soybean chromosomes viz., Chr.01, Chr.02, Chr.03, Chr.05, Chr.06, Chr.07, Chr.08, Chr.09, Chr.10, Chr.12, Chr.13, Chr.14, Chr.15, Chr.16, Chr.17, Chr.18 and Chr.20; no significant SNPs for the SPC were detected on the Chr.04, Chr.11 and Chr.19 (**Supplementary Table S4**). Distribution of these 59 SNPs across the different GWAS models and environments is presented in the UpSet plot (**Figures 5C, D**). We detected a maximum of eight SNPs on the Chr.18, and a minimum of one significant SNP on the chromosomes viz., Chr.07, Chr.10 and Chr.14; and the remaining 14 chromosomes possess the significant SNPs from two to six (**Supplementary Table S4**). The significant SNPs for the SPC were distributed across the different models as, the SNPs viz., Chr08_9054741 and Chr18_54408341 were detected by all the seven GWAS models, this is followed by SNP Chr18_54376853 that was detected by five models viz., GLM, MLMM, super, FarmCPU and BLINK; the remaining 56 SNPs were detected by one to three GWAS models (**Supplementary Table S4**). Moreover, the SNPs viz., Chr18_54408341 was detected by two single environments (CC22 & CC23) plus CE; and the SNPs viz., Chr01_11010539, Chr06_6762512 and Chr08_9054741 were detected by one single environment plus CE. The rest of the 55 SPC related SNPs were identified in one single environment or CE (**Supplementary Table S4**).

However, among the total of 105 and 59 SNPs detected to be associated with SOC and SPC, respectively; only three significant SNPs viz., Chr08_9054741, Chr08_9071236 and Chr08_9071263 were associated with both SOC and SPC (**Supplementary Table S4**). The effect of Chr08_9054741 was negative on both SOC (-1.34) and SPC (-2.04) traits, whereas the effect of Chr08_9071236 was positive on both SOC (1.35) and SPC (1.76); moreover, the effect of Chr08_9071263 was positive on SOC (1.32) as well as SPC (1.94). These results indicate that these three common SNPs govern the SOC and SPC traits in the same direction. The remaining SNPs were associated with either SOC or SPC (**Supplementary Table S4**).

### 3.4 Superior haplotypes for SOC and SPC

**Figure 6:**
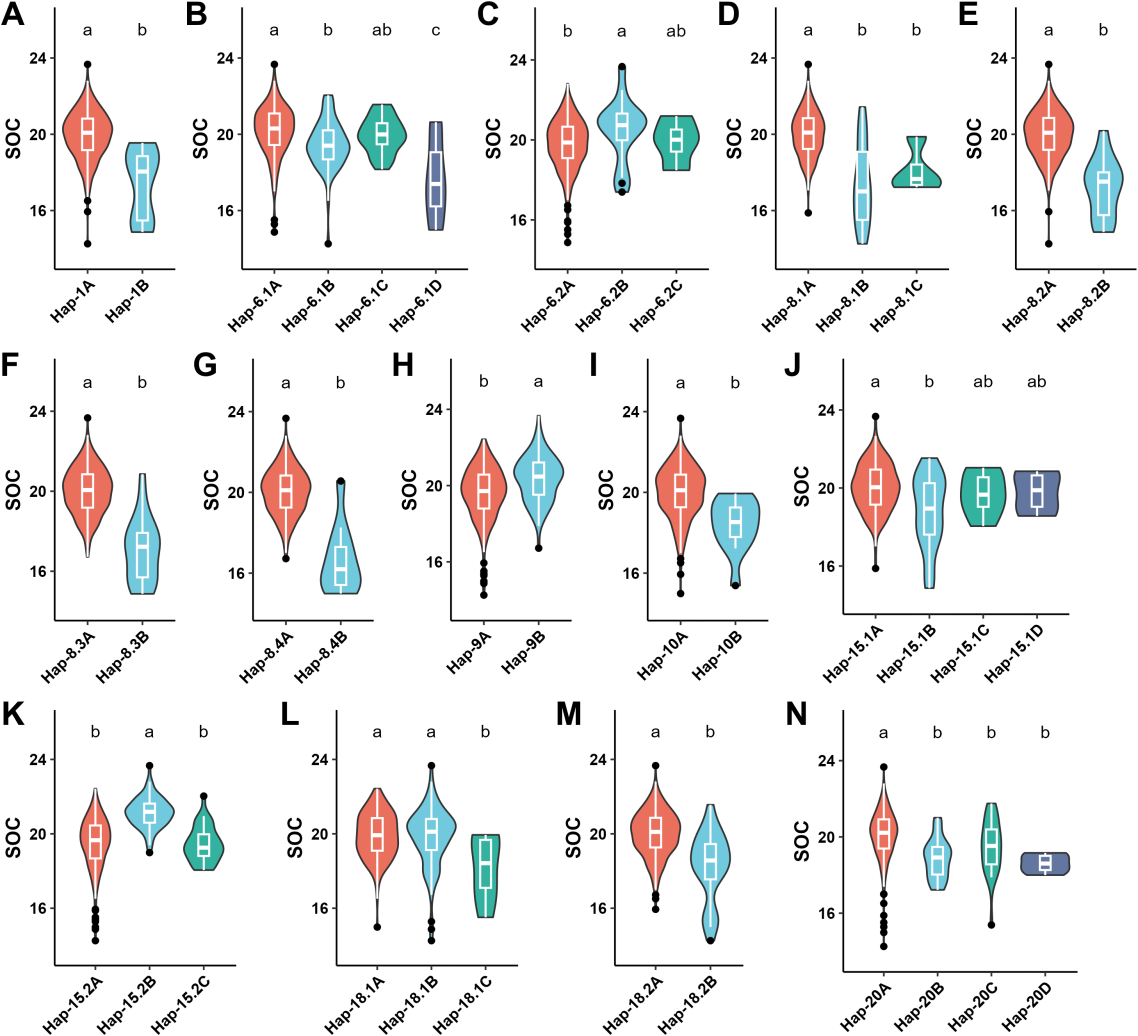
Analysis of 14 haplotype blocks associated with the SOC across the 340 accessions of soybean. **(A)** Hap-1, **(B)** Hap-6.1, **(C)** Hap-6.2, **(D)** Hap-8.1, **(E)** Hap-8.2, **(F)** Hap-8.3, **(G)** Hap-8.4, **(H)** Hap-9, **(I)** Hap-10, **(J)** Hap-15.1, **(K)** Hap-15.2, **(L)** Hap-18.2, **(M)** Hap-18.2, and **(N)** Hap-20. Haplotype allele number ranges from two to four across the 14 blocks in the soybean germplasm governing the SOC from the minimum to maximum levels through intermediate phenotypes. The different letters at the top of density curve correspond to differences at the significant level, whereas the same letters correspond to differences at the non-significant levels.

**Figure 7:**
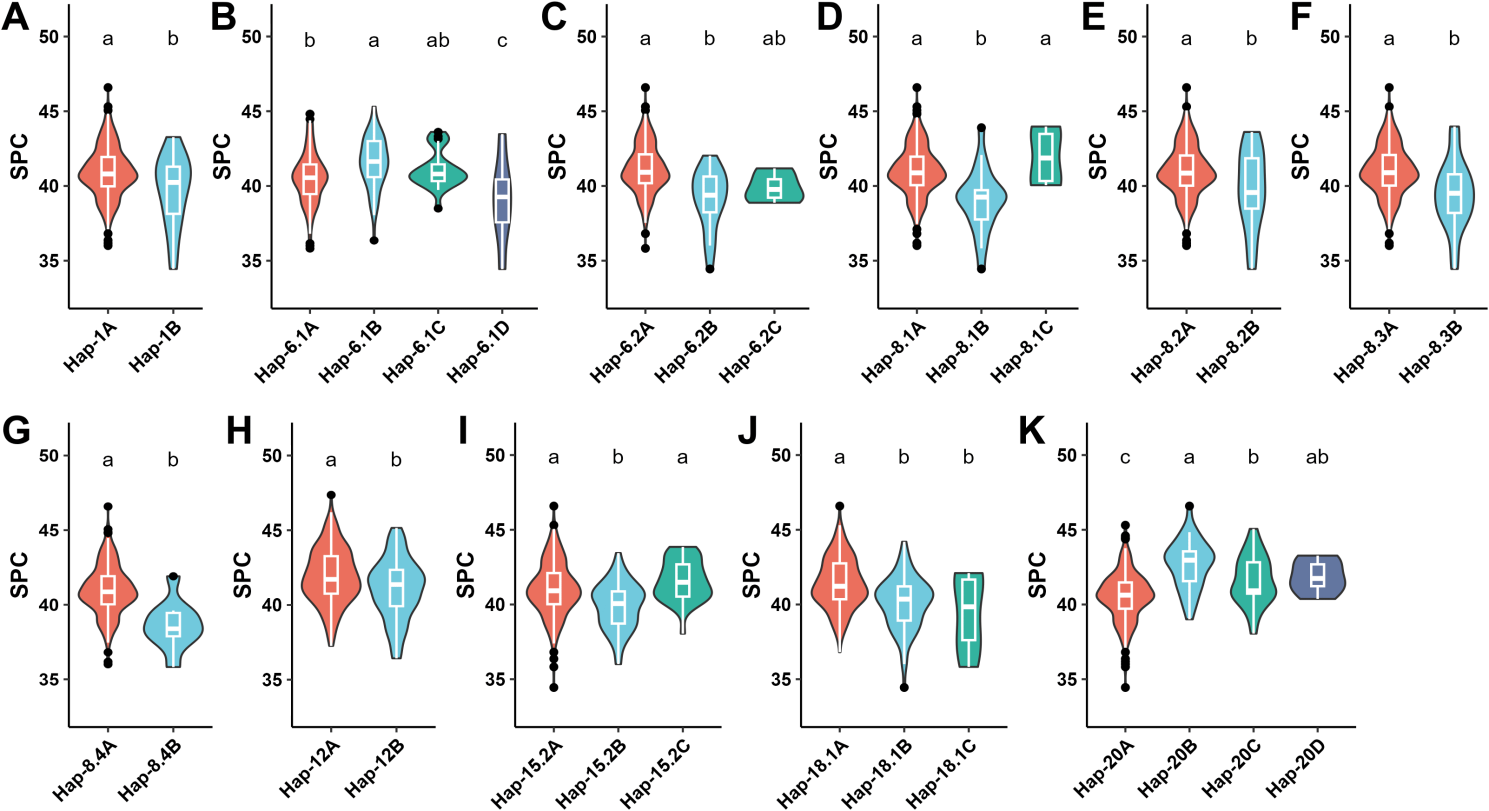
Analysis of 11 haplotype blocks associated with the SPC across the 340 accessions of soybean. **(A)** Hap-1, **(B)** Hap-6.1, **(C)** Hap-6.2, **(D)** Hap-8.1, **(E)** Hap-8.2, **(F)** Hap-8.3, **(G)** Hap-8.4, **(H)** Hap-12, **(I)** Hap-15.2, **(J)** Hap-18.1, and **(K)** Hap-20. Haplotype allele number ranges from two to four across the 11 blocks in the soybean germplasm governing the SPC from the minimum to maximum levels through intermediate phenotypes. The different letters at the top of density curve correspond to differences at the significant level, whereas the same letters correspond to differences at the non-significant levels.

The significant SNPs present in the same chromosome that were associated with the same trait or related traits, and were fitting into the LD decay range of ± 71.7 kb formed the haplotype blocks. Besides, the SNPs identified through different GWAS models but fell within the physical interval of ± 71.7 kb were also used for haplotype block construction. In this regard, our findings revealed that two, three, two, two, three, three, twenty-six, three, eleven, two, two, two, five, two and two significant SNPs associated with the SOC or/and SPC were present on the chromosomes viz., Chr.01, Chr.06, Chr.06, Chr.08, Chr.08, Chr.08, Chr.08, Chr.09, Chr.10, Chr.12, Chr.15, Chr.15, Chr.18, Chr.18 and Chr.20, respectively and are fitting into the half LD decay distance; therefore, they constituted 15 haplotype blocks viz., Hap-1, Hap-6.1, Hap-6.2, Hap-8.1, Hap-8.2, Hap-8.3, Hap-8.4, Hap-9, Hap-10, Hap-12, Hap-15.1, Hap-15.2, Hap-18.1, Hap-18.2 and Hap-20, respectively (**Figures 6**, **7**; **Supplementary Table S5**).

The number of haplotype alleles of these 15 haplotype blocks varied from two (Hap-1) to four (Hap-6.1) (**Figures 6**, **7**; **Supplementary Table S5**). Out of 15 haplotype blocks, the haplotype alleles of 14 blocks viz., Hap-1, Hap-6.1, Hap-6.2, Hap-8.1, Hap-8.2, Hap-8.3, Hap-8.4, Hap-9, Hap-10, Hap-15.1, Hap-15.2, Hap-18.1, Hap-18.2 and Hap-20 revealed differences at the significant level in the governing of SOC that varied from 16.68-21.15% (**Figure 6**; **Supplementary Table S5**). In contrast, the haplotype alleles of 11 out of total 16 haplotype blocks viz., Hap-1, Hap-6.1, Hap-6.2, Hap-8.1, Hap-8.2, Hap-8.3, Hap-8.4, Hap-12, Hap-15.2, Hap-18.1, and Hap-20 revealed differences at the significant levels in the governing of SPC that varied from 38.63-42.69%; whereas the haplotype alleles of the remaining four haplotype blocks viz., Hap-9, Hap-10, Hap-15.2, and Hap-18.1 showed differences at the non-significant levels in the governing of SPC (**Figure 7**; **Supplementary Table S5**).

In conclusion, the haplotype alleles of 14 and 11 haplotype blocks associated with SOC and SPC, respectively, regulate the different phenotypes of these two traits ranging from low-intermediate-high.

### 3.5 Stable QTL identification of SOC and SPC

Multiple significant SNPs were detected in different environments and models across the LD decay (± 71.7 kb) distance of 15 haplotype blocks viz., Hap-1, Hap-6.1, Hap-6.2, Hap-8.1, Hap-8.2, Hap-8.3, Hap-8.4, Hap-9, Hap-10, Hap-12, Hap-15.1, Hap-15.2, Hap-18.1, Hap-18.2 and Hap-20, hence they also represented the 15 stable QTLs named as *qSOC1*, *qSOC_SPC6.1*, *qSOC_SPC6.2*, *qSOC8.1*, *qSOC8.2*, *qSOC8.3*, *qSOC_SPC8*, *qSOC9*, *qSOC10*, *qSPC12*, *qSOC_SPC15.1*, *qSOC_SPC15.2*, *qSPC18*, *qSOC18* and *qSPC20* (**Supplementary Table S4**; **Supplementary Figure 2**). Besides, the genomic intervals of ± half LD-decay distance across the SNPs detected by various models and environments such as Chr01_11010539 and Chr15_10147967, which were present on Chr.01 and Chr.15, respectively, were defined as stable QTLs. They represent two QTLs referred to as *qSPC1* and *qSOC15*, respectively (**Supplementary Table S4**). Hence, together we identified a total of 17 QTLs; among these QTLs, the eight viz., *qSOC1*, *qSOC8.1*, *qSOC8.2*, *qSOC8.3*, *qSOC9*, *qSOC10*, *qSOC15* and *qSOC18* were associated with the SOC trait only, whereas the four QTLs viz., *qSPC1*, *qSPC12*, *qSPC18*, and *qSPC20* were associated with only SPC trait. However, the five QTLs viz., *qSOC_SPC6.1*, *qSOC_SPC6.2*, *qSOC_SPC8*, *qSOC_SPC15.1* and *qSOC_SPC15.2* were linked with both SOC and SPC traits (**Supplementary Table S4**; **Supplementary Figure 2**). The above genomic loci/QTLs were identified through various environments and GWAS models, that in turn indicates the persistency and stability of these genomic loci in the governing of SOC or/and SPC.

To quantify the genetic basis underlying the observed phenotypic correlation between SOC and SPC, we performed a linear regression analysis to evaluate the phenotypic variance explained (PVE) by the identified stable QTLs based on the phenotypic data from the CE (**Supplementary Table S6**). The five major pleiotropic QTL regions (*qSOC_SPC6.1*, *qSOC_SPC6.2*, *qSOC_SPC8*, *qSOC_SPC15.1* and *qSOC_SPC15.2*) were found to collectively account for 26.19% of the total variance for SOC and 22.26% for SPC. This indicated that around a quarter portion of the variation in both traits was governed by shared genomic “hotspots”. Furthermore, the other six stable QTLs of which the haplotype blocks exhibited significant individual effects on both traits (*qSOC1*, *qSOC8.1*, *qSOC8.2*, *qSOC8.3*, *qSPC18* and *qSPC20*) explained a total PVE of 22.17% for SOC and 29.49% for SPC. These 11 QTLs explained 48.36% of the phenotypic variance of SOC and 51.74% of SPC, respectively. The remaining phenotypic residuals (~31%) likely reflected the influence of numerous sub-threshold minor-effect loci and environmental factors. These results provide a genetic explanation for the significant correlation between SOC and SPC observed in our germplasm.

### 3.6 Candidate gene identification for SOC and SPC

**Figure 8:**
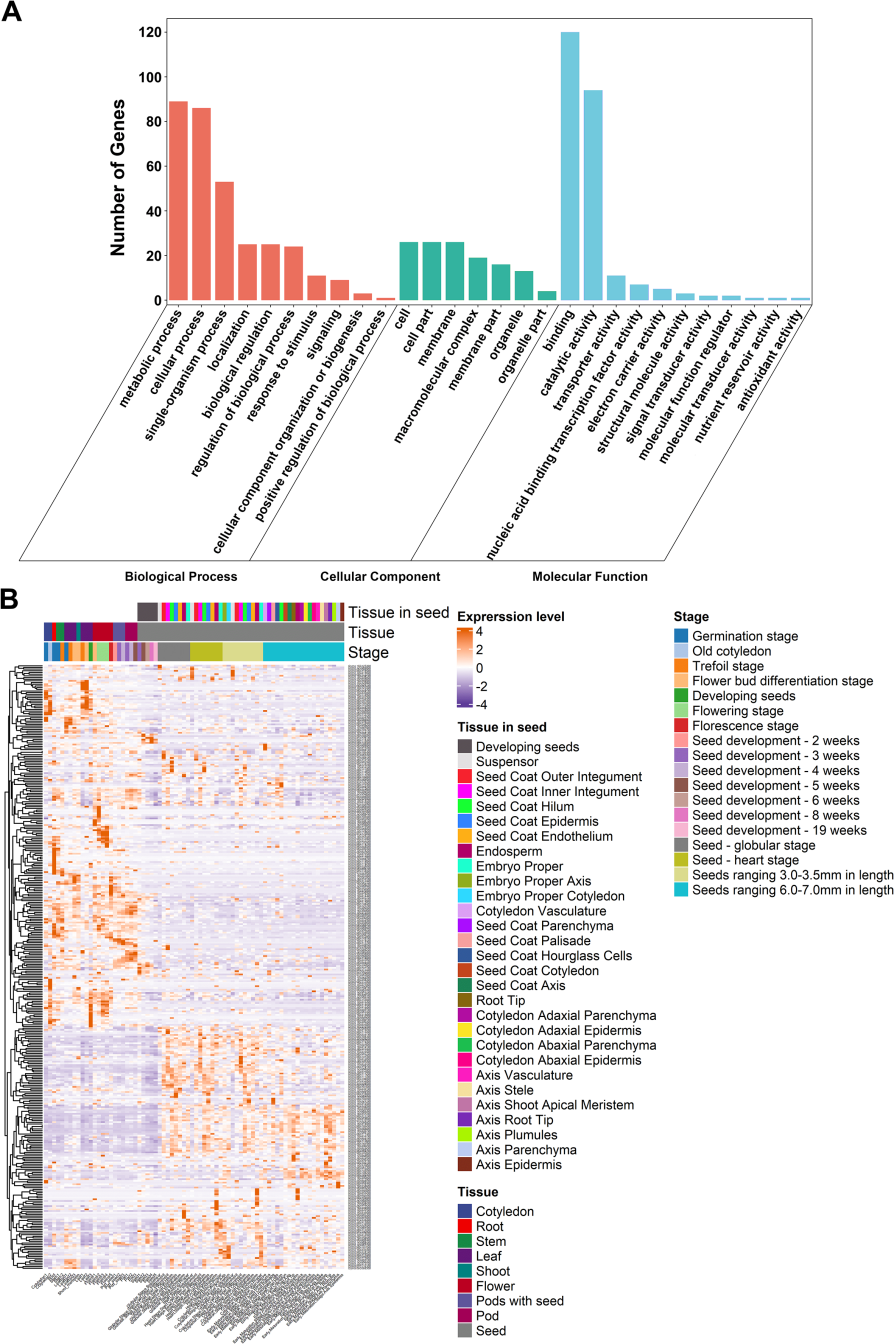
*In-silico* analysis of model genes occurring across the genomic interval of 17 QTLs. **(A)** This figure corresponds to gene ontology (GO) enrichment analysis of model genes for the terms belonging to three major categories viz., cellular component, biological process and metabolic function; and **(B)** This figure displays the expression pattern of the model genes present across the physical interval of 17 QTLs using the RNA-seq data; and the expression results are presented for the different development stages and tissues such as leaf, flower, pod and seed of soybean plant.

Here, we identified 353 model genes across the genomic intervals of 17 QTLs viz., *qSPC1*, *qSOC1*, *qSOC_SPC6.1*, *qSOC_SPC6.2*, *qSOC8.1*, *qSOC8.2*, *qSOC8.3*, *qSOC_SPC8*, *qSOC9*, *qSOC10*, *qSPC12*, *qSOC15, qSOC_SPC15.1*, *qSOC_SPC15.2*, *qSPC18*, *qSOC18* and *qSPC20*, that include 5, 15, 28, 24, 21, 19, 25, 41, 9, 30, 37, 13, 11, 22, 25, 14 and 14 genes, respectively. The gene ontology (GO) analysis was carried out by utilizing the online available web-based toolkit viz., agriGO V2.0; and this analysis showed the enrichment of various terms among the major categories viz., molecular function, biological process and cellular component within the 17 genomic loci linked with SOC and SPC traits (**Figure 8A**). The terms with the highest enrichment across the 17 QTLs are shown in **Figure 8A**. The gene expression levels of model genes residing in the physical intervals of 17 QTLs across the various tissues/organs and seed development stages of soybean are presented in the form of heatmap (**Figure 8B**). By using the *in-silico* analysis viz., function annotations, transcriptomic analysis and literature survey, we sorted out 121 genes across the genomic interval of 17 QTLs as possible candidates governing SOC and SPC traits in soybean (**Supplementary Table S7**). These include 3, 5, 11, 9, 11, 7, 7, 14, 3, 6, 5, 6, 4, 4, 8, 10, and 8 candidate genes across the physical interval of 17 QTLs viz., *qSPC1*, *qSOC1*, *qSOC_SPC6.1*, *qSOC_SPC6.2*, *qSOC8.1*, *qSOC8.2*, *qSOC8.3*, *qSOC_SPC8*, *qSOC9*, *qSOC10*, *qSPC12*, *qSOC15, qSOC_SPC15.1*, *qSOC_SPC15.2*, *qSPC18*, *qSOC18* and *qSPC20*, respectively (**Supplementary Table S7**). Gene function annotations such as amino acid biosynthesis, protein biosynthesis, amino acid and protein transport, fatty acid biosynthesis, lipid biosynthesis, fatty acid and lipid transport as well as processes related to the protein and lipid biosynthesis and their accumulation, and seed development (**Supplementary Table S7**), and genes showing higher expression (as showed by RNA-seq dataset) during the seed development stages were the main criteria to select the candidate genes (**Figure 8B**). These 121 genes were regarded as putative candidates for regulating the SOC and SPC in soybean.

To further screen out the genes regulating the SOC and SPC traits, we performed the sequence variation analysis, variant annotation analysis and haplotype analysis. Out of these 121 genes, only 104 genes showed sequence variations (**Supplementary Table S8**). Among these 104 genes, the haplotype alleles of 80 genes revealed differences at significant levels in governing the SOC or/and SPC (**Supplementary Tables S9**). In these 80 genes, the haplotype allele number varied from a minimum of two to the maximum of 8, regulating the SOC and SPC in the ranges of 15.98-21.23% and 37.69%-43.30%, respectively. Among these 80 genes, the haplotype alleles of 11 genes only showed differences at the significant levels in governing the SOC, whereas the haplotype alleles of 6 genes only showed differences at the significant levels in governing the SPC. However, in the remaining 63 genes (out of the total 80) the haplotype alleles revealed differences at the significant levels in governing of both SOC and SPC. The haplotype alleles of these 80 genes regulate the different phenotypes of SOC or SPC or both traits ranging from minimum to maximum levels through intermediate phenotypes. The variant annotation analysis showed that 45 of the 80 genes showed non-synonymous mutations, whereas the remaining 35 genes showed synonymous mutations (**Supplementary Table S8**). Hence, these 80 genes were prioritized as candidate genes governing the SOC and SPC traits in soybean (**Supplementary Table S9**).

**Figure 9:**
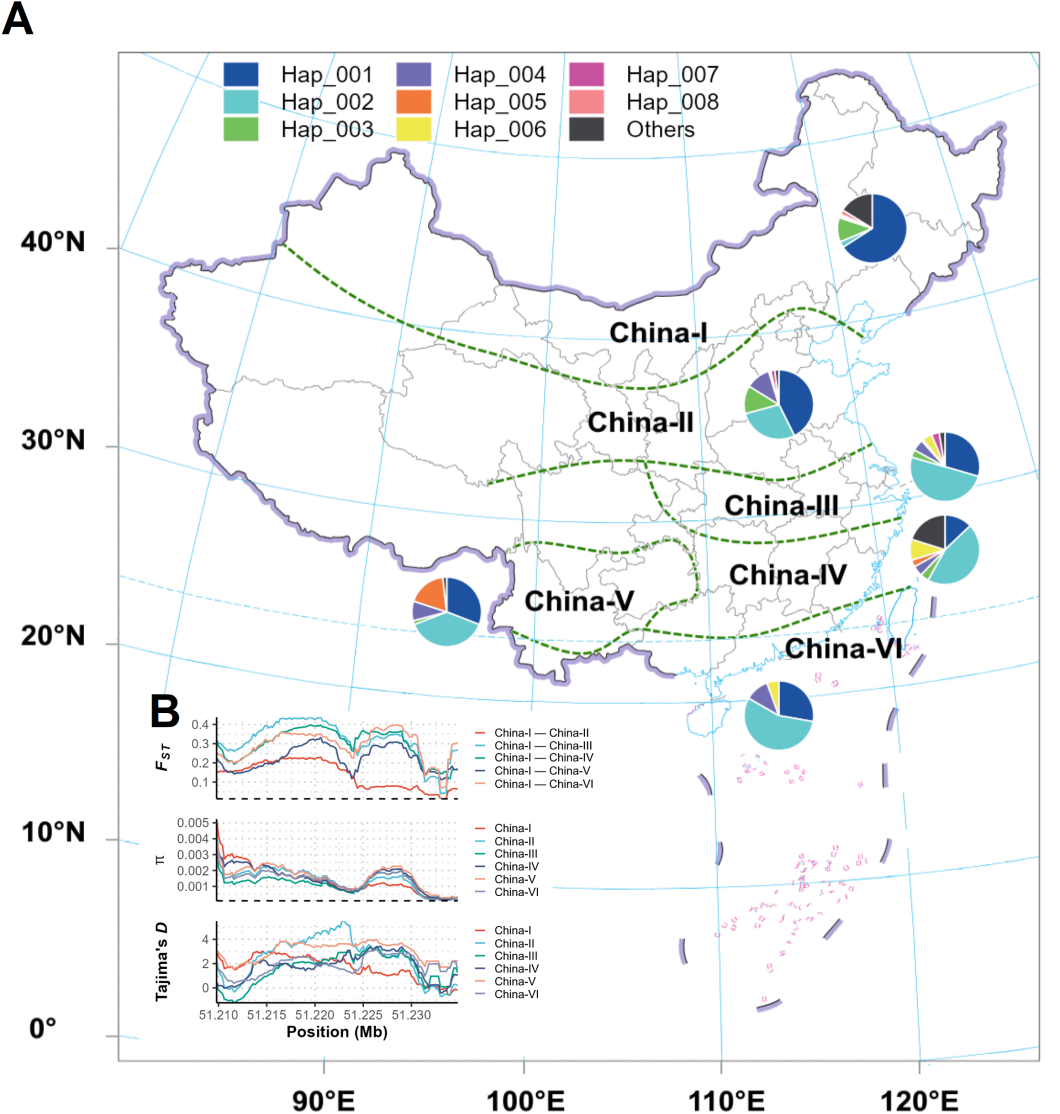
Distribution of different haplotypes of *Glyma.01G175500* across soybeans adapted to different regions. **(A)** Hap_001 was selected preferentially for the soybean adapted to low-latitudes, and Hap_002 was selected preferentially for the high-latitude adapted soybeans. **(B)***F_ST_*, *π* and Tajima’s *D* analysis revealing differential/divergent selection of *Glyma.01G175500* among the soybean populations adapted to different latitudes. The soybean production regions in China are divided into China-I, China-II, China-III, China-IV, China-V and China-VI following Liu et al. (2023).

Furthermore, we performed haplotype selection analysis of the soybean accessions adapted to different latitudes i.e., from low-latitude to high-latitude for these 80 genes using the public data from the SoyMD (https://yanglab.hzau.edu.cn/SoyMD/#/; **Supplementary Figures 3**-**8**). Our haplotype results showed that 12 genes viz., *Glyma.01G175500*, *Glyma.01G176000*, *Glyma.08G101100*, *Glyma.08G101900*, *Glyma.08G103100*, *Glyma.08G111700*, *Glyma.08G111800*, *Glyma.08G117000*, *Glyma.15G232200*, *Glyma.18G258000*, *Glyma.18G258100*, *Glyma.18G259100* underwent divergent selection for the specific haplotypes across the soybean population adapted to different latitudes (**Table 2**; **Figure 9**). For example, in *Glyma.01G175500*, the Hap_001 was predominantly selected in soybeans in Northeast China, whereas Hap_002 was predominantly selected in soybeans from southern parts of China. In the same way, the divergent specific haplotypes have been selected for the low- and high-latitude soybeans in the remaining 11 genes (**Table 2**). Moreover, the *F_ST_*, *π* and Tajima’s *D* analysis also revealed the differential/divergent selection of these 12 genes among the soybean populations adapted to different latitudes (**Supplementary Figures 3**-**8**). Hence, these 12 genes are the most potential candidates regulating the difference in the SOC and SPC traits in the soybeans adapted to the environment of Northeast China.

**Table 2:** Description of the 12 divergently selected genes across the different latitudes.

Gene	Orthologous gene in Arabidposis	Function annotation	Haplotypes selected in high-latitudes	Haplotype selected in low-latitudes
*Glyma.01G175500*	*AT3G50660.1 (CYP90B1);* Cytochrome P450 superfamily protein	Sterol biosynthetic process; unidimensional cell growth	Hap-001	Hap-002
*Glyma.01G176000*	*AT4G36630.1 (EMB2754);* Vacuolar sorting protein 39	Embryo development ending in seed dormancy; intracellular protein transport; protein N-linked glycosylation; protein glycosylation; protein targeting to vacuole	Hap-001	Hap-002
*Glyma.08G101100*	*AT1G67680.1;* SRP72 RNA-binding domain	SRP-dependent cotranslational protein targeting to membrane	Hap-001, Hap-003	Hap-002
*Glyma.08G101900*	*AT5G40780.1;* lysine histidine transporter 1	ER to Golgi vesicle-mediated transport; N-terminal protein myristoylation; amino acid import; amino acid transport; ammonium transport; anion transport; basic amino acid transport.	Hap-001	Hap-002
*Glyma.08G103100*	*AT1G24330.1;* ARM repeat superfamily protein	Protein ubiquitination	Hap-001, Hap-002	Hap-003, Hap-004
*Glyma.08G111700*	*AT5G55160.1 (SUM2/SUMO 2);* small ubiquitin-like modifier 2	Gluconeogenesis; methionine biosynthetic process; proteasomal protein catabolic process; protein sumoylation;	Hap-002	Hap-001
*Glyma.08G111800*	*AT5G55160.1 (SUM2/SUMO 2);* small ubiquitin-like modifier 2	Gluconeogenesis; methionine biosynthetic process; proteasomal protein catabolic process; protein sumoylation; response to heat	Hap-002	Hap-001, Hap-003
*Glyma.08G117000*	*AT5G45800.1 (MEE62);* Leucine-rich repeat protein kinase family protein	Embryo development ending in seed dormancy; protein phosphorylation; transmembrane receptor protein tyrosine kinase signaling pathway	Hap-001	Hap-002, Hap-003
*Glyma.15G232200*	*AT3G56710.1 (SIB1);* sigma factor binding protein 1	MAPK cascade; cellular response to nitrogen starvation; defense response, incompatible interaction.	Hap-002	Hap-001
*Glyma.18G258000*	*AT3G29590.1 (AT5MAT);* HXXXD-type acyl-transferase family protein	anthocyanin-containing compound biosynthetic process; response to UV-B; response to sucrose stimulus	Hap-001, Hap-003	Hap-002
*Glyma.18G258100*	*AT3G49190.1;* O-acyltransferase (WSD1-like) family protein	diacylglycerol O-acyltransferase activity	Hap-001	Hap-003, Hap-005
*Glyma.18G259100*	*AT3G28910.1 (ATMYB30, MYB3);* myb domain protein 30	response to auxin stimulus; very long-chain fatty acid biosynthetic process	Hap-001	Hap-002, Hap-005

## 4 Discussion

The SOC and SPC are the two crucial traits governing the quality and yield in soybean. Development of soybean cultivars with high SOC and SPC is the central objective of soybean researchers. However, it has been documented that soybeans in Northeast China have high SOC but low SPC compared to low-latitude soybeans (Rotundo et al., 2016; Guo et al., 2022). Little is known about the genetic mechanism regulating these differences in SOC and SPC traits in soybeans of Northeast China. Hence, there is a need to explore the genetic mechanism of these differences across the soybeans adapted to different latitudes. The amount of SOC and SPC in the global soybean germplasm varies from 8.10-27.10% and 34.1–56.8%, respectively (Wang et al., 2025). In agreement, our study also showed that SOC and SPC varied from 13.59-24.67% and 31.72-50.83%, respectively in the Northeast China adapted soybean germplasm. Hence, a greater emphasis is needed to be put on the characterization of soybean germplasm for the SOC and SPC traits. The approaches of classical breeding have been earlier used to enhance the SOC and SPC in soybean; however, it has been documented that these breeding approaches have developed soybean cultivars with high SOC but at the cost of reduced SPC and vice versa (Karikari et al., 2019). In agreement, our study also reported a significant negative correlation between SOC and SPC (**Figure 1C**). The significant negative correlation between SOC and SPC has hindered the simultaneous improvement of these two traits in the same cultivar. Furthermore, the polygenic inheritance and environmental sensitiveness of the SOC and SPC traits make the conventional breeding unable to meet the demands (Duan et al., 2023). In this context, molecular breeding has promised to develop soybean cultivars with both high SOC and SPC especially by using the independent or non-correlated QTLs/genes (Patil et al., 2018; Moore Ronald et al., 2025). Hence, identifying the independent MTAs/QTLs/genes for these two traits, and their subsequent deployment in the breeding programs of soybean will break the negative correlation between SOC and SPC; thereby producing the soybean cultivars with both high SOC and SPC which is the long-term goal of soybean breeders. Hence, approaches of molecular breeding have increased ability and precision to develop improved cultivars in soybean (Duan et al., 2025). Here, we utilized combined strategy of GWAS, haplotype analysis, QTL analysis, and candidate gene analysis to elucidate the comprehensive genetic basis of SOC and SPC traits in soybeans in Northeast China.

### 4.1 Phenotypic variability of the GWAS population

Here, a set of 340 diverse accessions of soybean was assessed for SOC and SPC traits at two locations viz., Changchun and Jiutai research farms, corresponding to the entire of four environments viz., CC22, CC23, CC24 and JT24 plus CE. The effects of *G*, *E* and *G* × *E* interactions on the SOC and SPC traits were highly significant, which is in accordance with the findings documented by earlier authors (Li et al., 2019; Jin et al., 2023). These results in turn indicated the great potential of this soybean germplasm for the improvement of these traits; besides, the differences in the environmental variables among various environments have resulted in the different trait values of the SOC and SPC in the same set of soybean accessions. The higher value of *H* for the SOC and SPC traits indicated the same accessions of GWAS panel will perform alike if the same environmental conditions were provided. In agreement, many previous studies have reported similar outcomes for the SOC and SPC traits in soybean (Li et al., 2019; Diers et al., 2023; Kim et al., 2023). Moreover, highly significant *G*, *E* and *G × E* interactions shown by SOC and SPC revealed their complex pattern of inheritance; and similar outcomes are reported by earlier researchers (Jin et al., 2023).

### 4.2 Dissecting the genetic basis of SOC and SPC traits using the GWAS approach

In recent years, GWAS has been routinely used in plant research for the elucidation of genetic basis of different traits; and the special thanks go to the progress done in genome sequencing and high-throughput genotyping (Alqudah et al., 2020). In soybean, GWAS is utilized to explore the genetic elements of multiple traits including the SOC and SPC (Lee et al., 2019; Li et al., 2019; Jin et al., 2023). Hence, the current study used the advanced SNP genotyping in the GWAS approach to unravel the genomic loci and genes for SOC and SPC traits in soybean. Here, we detected the significant association of 105 and 59 SNPs with the SOC and SPC, respectively across the multiple environments (CC22, CC23, CC24 & JT24) plus CE and seven GWAS models. These 105 and 59 significant SNPs of SOC and SPC were located on the 18 and 17 chromosomes of soybean, respectively, indicating multigenic inheritance of both traits, i.e. regulated by multiple genes. In accordance with our findings, the previous research studies have also documented the similar results of SOC and SPC traits in soybean (Jin et al., 2023; Doszhanova et al., 2024). Among the 105 and 59 SNPs detected significantly associated with SOC and SPC traits, respectively; only three common significant SNPs were linked with both SOC and SPC viz., Chr08_9054741, Chr08_9071236 and Chr08_9071263, while the remaining significant SNPs were either associated with the SOC or SPC. The three common SNPs associated with both SOC and SPC traits indicated their pleiotropic effect on the two traits, that may be because these SNPs were associated with genes encoding the enzyme involved in the common metabolic pathways (Gao et al., 2024; Van et al., 2025).

Furthermore, our outcomes revealed substantial variation in the identification of significant SNPs by seven different models for both traits. For instance, the Chr08_9071263 linked with SOC was detected by all seven models; in comparison, only one GWAS model detected the 30 significant SNPs. Similarly, the significant SNPs viz., Chr08_9054741 and Chr18_54408341 of SPC were detected by all seven GWAS models, whereas 27 SNPs of SPC were detected by a single GWAS model. Similar results have been reported by the earlier studies for various crop plants including soybean (Leventhal et al., 2025), maize (Kaler et al., 2020) and wheat (Merrick et al., 2022). The reason to describe this phenomenon is because the various models of GWAS are built on divergent hypotheses involving different distribution characteristics of QTL effects; hence, this leads to the difference in SNP detection among various GWAS models (Odell et al., 2022).

The moderate negative phenotypic correlation between SOC and SPC might be explained at the genetic level because some of the SNPs, QTLs and haplotype blocks affecting both traits simultaneously. Besides, the effects of environment as well as *G* × *E* were highly significant in the GWAS population that might have further resulted in the negative correlation. The correlation between SOC and SPC was underpinned by a substantial shared genetic basis, as evidenced by the collective PVE of approximately 26% and 22% by the major pleiotropic QTLs, respectively (**Supplementary Table S6**). Considering the other six stable QTLs of which the haplotype blocks exhibited significant individual effects on both traits, the 11 detected QTLs explained 56.23% of the heritability of SOC and 65.50% of SPC, respectively. Our regression analysis showed that the detected stable QTLs have already explained more than half of the genetic commonality. This implies that the observed phenotypic linkage is not merely a localized phenomenon but the aggregate result of multiple stable genetic regions.

Besides, substantial variation has been observed for the SNPs detected for both traits across the different environments. In some genomic regions, a locus may surpass the significance threshold for one trait/environment while having a significance level below the threshold for the other one. These suggestive signals may show consistent peak patterns across both traits/environments, supporting shared genetic control, but might be masked by statistical corrections and *G* × *E* interactions. These outcomes revealed that SOC and SPC traits are substantially influenced by the environment, hence supporting the highly significant *G* × *E* reported in this study.

### 4.3 Superior haplotypes identified for optimizing the SOC and SPC in soybean

In the GWAS analysis, soybean researchers have mostly used biallelic SNP markers; and these biallelic markers are not able to detect the multiple alleles present across the diverse GWAS panel that might represent the superior and rare alleles, hence making this valuable genetic diversity unavailable for the crop improvement (He and Gai, 2023). Therefore, there is an important need for multiallelic markers in the GWAS analysis to trap these superior/rare alleles as well as non-linear variation across the diverse natural crop germplasm. In this context, multiallelic markers of haplotypes have promised to fix these challenges of biallelic SNP markers in the GWAS (Bhat et al., 2021; Voorrips and Tumino, 2022). Hence, these markers will make the considerable genetic diversity accessible for crop improvement (Voorrips and Tumino, 2022). In the last few years, some studies have identified the superior haplotypes for various traits in soybean such as traits related to yield (Shao et al., 2022), plant height (Qin et al., 2023) and salinity tolerance (Patil et al., 2016). Besides, superior haplotypes are also detected in other crop species such as in pigeonpea for water deficit tolerance (Sinha et al., 2020) and in rice for quality traits (Abbai et al., 2019). There are multiple mechanisms including selection, recombination and mutation that govern the haplotype variation across the diverse crop germplasm (Hellsten et al., 2013). Hence, identifying and utilizing the haplotypes in molecular breeding has a substantial impact in crop improvement (Sinha et al., 2020). Here, we identified a total of 15 haplotype blocks, and the haplotype alleles of 14 out of total 15 haplotype blocks showed differences at the significant levels in the governing of SOC, whereas among the 15 haplotype blocks, the haplotype alleles of 11 blocks revealed differences at the significant levels in the governing of SPC. The haplotype alleles of these 15 blocks vary from two to four regulating the SOC or SPC from the low-intermediate-high levels. Among the 15 haplotype blocks, the haplotype alleles of Hap-1, Hap-8.2, Hap-8.3, Hap-8.4, showed positive correlation in the regulation of SOC and SPC; whereas the haplotype alleles of Hap-6.2, Hap-15.2 and Hap-20 showed negative correlation in the regulation of the SOC and SPC traits. However, the haplotype alleles of Hap-9, Hap-10, Hap-15.1, Hap-18.2 showed non-significant differences on the regulation of SPC, but revealed differences at the significant level in the governing of SOC; whereas the haplotype alleles of Hap-12 revealed differences at the significant level in the governing of SPC but revealed differences at the non-significant level in the governing of SOC. These findings suggest the SOC and SPC traits of soybean possess complicated genetic structure, where some genetic loci have positive effects on both SOC and SPC traits, and some genetic loci showed negative correlations in the regulation of SOC and SPC; and some loci have independent effects on either SOC or SPC. Our study showed that alleles of each haplotype block regulate the varied phenotypes of SOC and SPC, thus delivering an opportunity to optimize the desired quantity of SOC and SPC in the soybean cultivar. These haplotypes can be used to produce cultivars of soybean with optimized as well as improved SOC and SPC, hence will substantially impact the quality and yield of soybeans.

### 4.4 Stable QTLs detected for SOC and SPC traits

By considering the genomic regions (± half LD-decay distance) flanking the two significant SNPs detected through various models and environments as well as the LD decay distance across the 15 identified haplotype blocks, we delineated a total of 17 stable QTLs associated with SOC or SPC or both traits. Among these 17 QTLs, 11 of them fell within the genomic interval of previously reported QTLs, hence not being considered as novel QTLs (**Supplementary Figure 2**). For example, Zhang et al. (2025) identified the significant SNP associated with SPC on Chr.06, and that SNP fell within the genomic interval of *qSOC_SPC6.1* (5133255–5365962 bp). The *qSOC_SPC6.2* present on Chr.06 was present in the genomic regions of earlier reported QTLs viz., cqSeed protein-015 (5449370–7326665 bp) and cqSeed oil-016 (5504147–8260733 bp) (Pathan et al., 2013). Hence, the *qSOC_SPC6.2* might belong to the same cqSeed protein-015 and cqSeed oil-016 QTLs, as previously reported by Pathan et al. (2013). The *qSOC8.1* fell within the genomic region (7525742- 9439751) of previously reported QTL viz., Seed oil 11-g2 (Yao et al., 2020). Similarly, *qSOC8.2* fell within the genomic regions (7966391- 11892640) of earlier reported QTL viz., Seed oil 1-1 (Mansur et al., 1993). The physical interval of *qSOC8.3* was located within the genomic region of earlier reported QTL viz., Seed oil 43-1 (Mao et al., 2013). The *qSOC_SPC8* present on the Chr.08 fell within the physical intervals of earlier reported QTLs viz., Seed oil 8-g13 (Zhang et al., 2018b) and Seed protein 34-5 (Lu et al., 2013) QTLs, hence *qSOC_SPC8* might be the same QTLs as that of Seed oil 8-g13 and Seed protein 34-5. The *qSOC10* present on the Chr.10 was located within the physical interval (44580197- 44718071) of earlier reported QTL viz., Seed oil 39-16, hence representing the same QTL (Wang et al., 2014). Moreover, the *qSPC12* fell within the genomic region (4520131- 6391062) of previously reported QTL viz., Seed protein 28-3 (Liang et al., 2010). *qSOC15* located on the Chr.15 might be the same QTLs as previously reported mqSeed Oil-014 (Qi et al., 2011), as the *qSOC15* fell within the genomic interval (10392903-10393032) of mqSeed Oil-014. The *qSPC18* lied across the genomic region (1736692-56797602) of earlier documented QTL viz., Seed protein 36-25 (Mao et al., 2013). The *qSPC20* fell within the genomic interval (2615587–41000124 bp) of previously reported QTL viz., Seed protein 36-26 (Mao et al., 2013). Zhao et al (2024) found that the SNP named rs20739 present on Chr.18 was associated with SOC, which fell within the genomic interval of *qSOC18* (57712803-57856803). However, no QTL was identified to date that lies across the genomic interval of *qSOC1*, *qSPC1*, *qSOC9*, *qSOC_SPC15.1* and *qSOC_SPC15.2* (**Supplementary Figure 2**). Therefore, these five QTLs were newly reported/novel QTLs for SOC or/and SPC identified in the current study. Here, we report that GWAS analysis substantially diminished the genomic interval of the earlier detected QTLs, thus providing the proof for the higher resolution of GWAS analysis in QTL identification. The classical mapping method possesses reduced resolution because it utilizes the biparental population in the genetic mapping, that in turn results in a larger confidence interval of earlier documented QTLs (Yu et al., 2024). Hence, high-resolution stable QTLs identified by the GWAS in the current study will be potentially harnessed in the molecular breeding programs for the improvement of SOC and SPC in soybean.

### 4.5 Identifying potential candidate genes regulating SOC and SPC in soybean

The central objective of the researcher is to find-out true candidate genes governing the trait of interest that are underlying the major QTLs. Successful validation of function for the candidate genes underlying the QTLs will dictate their potential use in breeding programs. The true genes regulating the SOC and SPC traits have remained largely unexplored, and only a few genes regulating these traits in soybean have been characterized (as reviewed by Mo et al. (2024)). Based on the *in-silico*, sequence variation, variant annotation and haplotype analysis, we prioritized 80 genes as the candidates regulating the SOC and SPC in Northeast China soybeans. Among them, 12 genes undergo divergent selection between low-latitudes and high-latitudes; hence, these 12 genes will be considered as the potential candidates regulating SOC and SPC traits in soybean, as well as regulating the difference in the quality traits among the soybeans adapted to different environments. For example, the ortholog of *Glyma.01G175500* in *Arabidopsis thaliana* is *CYP90B1* (Zhang et al., 2024a). The *CYP90B1* encodes the steroid C-22 hydroxylase that plays an important role in the sterol biosynthesis (Zhang et al., 2024a). The ortholog of *Glyma.01G176000* is *EMB2754*, and the *EMB2754* encodes the Vacuolar sorting protein 39, and has been reported to transport the proteins into vacuoles during seed development (Tsao et al., 2022), and lipid transfer in Arabidopsis (Michaud et al., 2017). The *Glyma.08G101100* ortholog in Arabidopsis is *AT1G67680* that encodes SRP72; the SRP protein mediates the transport of secretory proteins to the endoplasmic reticulum (ER) in plants, and it has been documented that storage proteins reach the protein storage vacuoles (PSV) through a vesicle-mediated biosynthetic trafficking pathway that includes the ER, the Golgi apparatus, the tubulovesicular network (TGN) and the endosomes/prevacuoles (Zeng et al., 2023). *Glyma.08G101900* is an ortholog of *AT5G40780* which encodes lysine histidine transporter 1 (LHT1) in Arabidopsis (Chen and Bush, 1997). The LHT1 is a high-affinity transporter for cellular amino acids in both roots and shoots, hence is important in the biosynthesis of proteins (Rabby et al., 2022). In soybean the *Glyma.08G103100* is an ortholog of *AT1G24330* which encodes the ARM repeat superfamily protein; and this protein belongs to E3 ubiquitin ligase. In Brassica species the ARM repeat protein viz., ARC1 acts downstream of the S receptor kinase to promote ubiquitination and protein degradation (Stone et al., 2003). The *Glyma.08G111700* and *Glyma.08G111800* are the ortholog of *AT5G55160/SUMO2/ATSUMO2* which encodes for small ubiquitin-like modifier 2 in Arabidopsis (**Table 2**). The ubiquitin-like modifier stabilizes the proteins to which it is conjugated; and removal of the ubiquitin-like modifier results in degradation of the proteins (Miura et al., 2009). Gene viz., *Glyma.08G117000* is an ortholog of *AT5G45800*/*MEE62* that encodes for leucine-rich repeat protein kinase family protein, and these proteins have been documented to play important roles in plant growth, development and stress responses (Song et al., 2022). The *Glyma.15G232200* is an ortholog of *AT3G56710/SIB1* gene that encodes for sigma factor binding protein 1; and this gene has been documented to play an important role under nitrogen starvation, and nitrogen is the primary component in the protein synthesis (Ramel et al., 2007). The *Glyma.18G258000* and *Glyma.18G258100* are the orthologs of *AT3G29590/AT5MAT* and *AT3G49190*, respectively that encode for acyl-transferase family proteins, and these proteins are important in the *de-novo* fatty-acid biosynthesis (Yang et al., 2021). Moreover, the ortholog of *Glyma.18G259100* in *Arabidopsis thaliana* is *AT3G28910/MYB30* (Liu et al., 2014; Yan et al., 2020). The MYB30 belongs to subgroup I, that governs the biosynthesis of very-long-chain fatty acids (VLCFAs) by regulating genes encoding the four enzymes of the acyl-CoA elongase complex (Zhukov and Popov, 2022). Hence, the divergent selection of these 12 genes across the soybean populations adapted to different regions might be the reason of the unique characteristics of the SOC and SPC in Northeast China.

In conclusion, we identified the haplotype alleles within the 15 haplotype blocks as well as within the 80 candidate genes across the soybean germplasm of 340 accessions that regulate the SOC or SPC or both traits from the minimum to maximum levels through different intermediate phenotypes. Hence, these haplotypes can be used to produce cultivars of soybean with desired SOC and SPC.

Besides, we identified key genes that were divergently selected between the soybeans adapted to different regions of China, and they might be the important genes regulating the difference in the SOC and SPC across the various latitude regions.

## 5 Conclusion

Here, we utilized a comprehensive approach to find-out the genetic structure of SOC and SPC traits in high-latitude adapted soybeans. The present study identified 105 and 59 significant SNPs associated with the SOC and SPC traits, respectively across the germplasm; and among them only three significant SNPs were associated with both SOC and SPC. Besides, we identified a total of 15 haplotype blocks distributed on nine chromosomes. Among them 14 and 11 haplotype blocks were linked with SOC and SPC, respectively. Furthermore, the present study identified 17 QTLs associated with either SOC or SPC or both traits; five of them were novel identified for the first time. *In-silico* analyses allowed us to prioritize 121 putative candidate genes present across the genomic regions of the 17 identified QTLs. Furthermore, based on the sequence variation, variant annotation, and haplotype analysis, 80 of the 121 genes were screened out as potential candidates regulating the SOC and/or SPC traits. In these 80 genes, the haplotype allele number varied from 2–8 across soybean germplasm of 340 accessions. Overall, the haplotype alleles identified in the 15 haplotype blocks and 80 candidate genes across the 340 accessions regulate the SOC or/and SPC from the minimum to maximum levels through intermediate phenotypes. Furthermore, our results revealed that 12 of these 80 genes were divergently selected below low- and high-latitudes; hence they might be the major genes regulating the difference in the SOC and SPC traits in the low-latitudes and high-latitudes. We identified many independent SNPs, haplotypes and QTLs associated with either SOC or SPC that can provide the opportunity to improve these two highly negatively-correlated traits simultaneously in the same soybean cultivar. Overall, the insights generated from this study provide valuable genomic resources for the optimization of the SOC and SPC in the soybean cultivars. In conclusion, the novel findings reported in this study hold significant potential for developing Northeast-China adapted soybean cultivars with enhanced quality and yield.

## Data Availability

The datasets presented in this study can be found in online repositories. The names of the repository/repositories and accession number(s) can be found below: https://ngdc.cncb.ac.cn/gvm/, GVM000764.

## Supplementary material

Supplementary Figure 1 Frequency distribution of the SOC **(A)** and SPC **(B)** in the GWAS panel of 340 soybean accessions across the four different environments viz., CC22, CC23, CC24 & JT24.

Supplementary Figure 2 Map showing the comparison of the QTLs identified in the present study with the previously reported QTLs.

Supplementary Figure 3 Diagram showing the *F_ST_*, *π* and Tajima’s *D* analysis of population differentiation as well as distribution of the different haplotypes of *Glyma.01G175500* and *Glyma.01G176000* genes across the soybeans adapted to different latitudes (China-I to China-VI).

Supplementary Figure 4 Diagram showing the *F_ST_*, *π* and Tajima’s *D* analysis of population differentiation as well as distribution of the different haplotypes of *Glyma.08G101100*, *Glyma.08G101900* and *Glyma.06G103100* genes across the soybeans adapted to different latitudes (China-I to China-VI).

Supplementary Figure 5 Diagram showing the *F_ST_*, *π* and Tajima’s *D* analysis of population differentiation as well as distribution of the different haplotypes of *Glyma.08G111700*, and *Glyma.06G111800* genes across the soybeans adapted to different latitudes (China-I to China-VI).

Supplementary Figure 6 Diagram showing the *F_ST_*, *π* and Tajima’s *D* analysis of population differentiation as well as distribution of the different haplotypes of *Glyma.08G117000*, across the soybeans adapted to different latitudes (China-I to China-VI).

Supplementary Figure 7 Diagram showing the *F_ST_*, *π* and Tajima’s *D* analysis of population differentiation as well as distribution of the different haplotypes of *Glyma.15G232200* across the soybeans adapted to different latitudes (China-I to China-VI).

Supplementary Figure 8 Diagram showing the *F_ST_*, *π* and Tajima’s *D* analysis of population differentiation as well as distribution of the different haplotypes of *Glyma.18G258000*, *Glyma.18G258100* and *Glyma.18G259100* genes across the soybeans adapted to different latitudes (China-I to China-VI).

Supplementary Table 1 List of 340 soybean accessions along with their geographic origin used in the GWAS analysis.

Supplementary Table 2 Phenotypic analysis of seed oil content (SOC) and seed protein content (SPC) of 340 soybean accessions evaluated in different individual environments plus combined environment (CE).

Supplementary Table 3 Distribution of the SNPs used for GWAS across soybean chromosomes.

Supplementary Table 4 SNP markers and stable QTLs significantly associated with seed oil content (SOC) and seed protein content (SPC) across different environments and models.

Supplementary Table 5 Haplotype analysis of 15 haplotype blocks identified in the present study across the panel of 340 soybean accessions.

Supplementary Table 6 Phenotypic variance explained (PVE) by the identified 17 stable QTLs in the combined environment.

Supplementary Table 7 Putative candidate genes selected based on the *in-silico* analysis and gene function annotation across the 17 QTLs.

Supplementary Table 8 Variant annotation of selected putative candidate genes (only 104 among the total 121 genes selected based on the *in-silico* analysis and gene function annotation showed variant annotation).

Supplementary Table 9 The haplotype analysis results of the candidate genes.
