# Supplementary material for: Genome-wide detection of superior haplotypes for seed oil and protein content in Northeast China soybean (Glycine max L.) germplasm
Source: Front Plant Sci. 2026 Feb 24;17:1767299. doi: 10.3389/fpls.2026.1767299 (PMC12971530; doi:10.3389/fpls.2026.1767299)
Supplement: Supplementary Figure 1 — Frequency distribution of the SOC (A) and SPC (B) in the GWAS panel of 340 soybean accessions across the four different environments viz., CC22, CC23, CC24 & JT24. [file DataSheet1.docx]

Supplementary Material





**Supplementary Figure 1.** Frequency distribution of the SOC (A) and SPC (B) in the GWAS panel of 340 soybean accessions across the four different environments viz., CC22, CC23, CC24 & JT24.





**Supplementary Figure 2.** Map showing the comparison of the QTLs identified in the present study with the previously reported QTLs. Diagram showing six QTLs viz., *qSOC1*, *qSPC1*, *qSOC9*, *qSOC_SPC15.1* and *qSOC_SPC15.2* are identified for the first time, and the rest of the 12 QTLs viz., *qSOC_SPC6.1*, *qSOC_SPC6.2*, *qSOC8.1*, *qSOC8.2*, *qSOC8.3*, *qSOC_SPC8*, *qSOC10*, *qSPC12*, *qSOC15*, *qSPC18* , *qSOC18* and *qSPC20* are located within the genomic region of previously reported QTLs.





**Supplementary Figure 3.** Diagram showing the *F_ST_*, *π* and Tajima’s *D* analysis of population differentiation as well as distribution of the different haplotypes of *Glyma.01G175500* and *Glyma.01G176000* genes across the soybeans adapted to different latitudes (China-I to China-VI).





**Supplementary Figure 4.** Diagram showing the *F_ST_*, *π* and Tajima’s *D* analysis of population differentiation as well as distribution of the different haplotypes of *Glyma.08G101100*, *Glyma.08G101900* and *Glyma.06G103100* genes across the soybeans adapted to different latitudes (China-I to China-VI).





**Supplementary Figure 5.** Diagram showing the *F_ST_*, *π* and Tajima’s *D* analysis of population differentiation as well as distribution of the different haplotypes of *Glyma.08G111700*, and *Glyma.06G111800* genes across the soybeans adapted to different latitudes (China-I to China-VI).





**Supplementary Figure 6.** Diagram showing the *F_ST_*, *π* and Tajima’s *D* analysis of population differentiation as well as distribution of the different haplotypes of *Glyma.08G117000*, across the soybeans adapted to different latitudes (China-I to China-VI).





**Supplementary Figure 7.** Diagram showing the *F_ST_*, *π* and Tajima’s *D* analysis of population differentiation as well as distribution of the different haplotypes of *Glyma.15G232200* across the soybeans adapted to different latitudes (China-I to China-VI).





**Supplementary Figure 8.** Diagram showing the *F_ST_*, *π* and Tajima’s *D* analysis of population differentiation as well as distribution of the different haplotypes of *Glyma.18G258000*, *Glyma.18G258100* and *Glyma.18G259100* genes across the soybeans adapted to different latitudes (China-I to China-VI).

**Supplementary Table S1.** List of 340 soybean accessions along with their geographic origin used in the GWAS analysis.

| **ID** | **Geographic origin** |
| --- | --- |
| FENGGWS001 | Other region of China |
| FENGGWS004 | Heilongjiang, China |
| FENGGWS005 | Heilongjiang, China |
| FENGGWS006 | Heilongjiang, China |
| FENGGWS007 | Heilongjiang, China |
| FENGGWS008 | Heilongjiang, China |
| FENGGWS009 | Other region of China |
| FENGGWS011 | Heilongjiang, China |
| FENGGWS012 | Heilongjiang, China |
| FENGGWS013 | Heilongjiang, China |
| FENGGWS014 | Heilongjiang, China |
| FENGGWS015 | Heilongjiang, China |
| FENGGWS016 | Heilongjiang, China |
| FENGGWS017 | Heilongjiang, China |
| FENGGWS018 | Heilongjiang, China |
| FENGGWS019 | Heilongjiang, China |
| FENGGWS020 | Heilongjiang, China |
| FENGGWS021 | Heilongjiang, China |
| FENGGWS022 | Heilongjiang, China |
| FENGGWS023 | Heilongjiang, China |
| FENGGWS024 | Heilongjiang, China |
| FENGGWS025 | Heilongjiang, China |
| FENGGWS026 | Heilongjiang, China |
| FENGGWS027 | Heilongjiang, China |
| FENGGWS028 | Heilongjiang, China |
| FENGGWS029 | Heilongjiang, China |
| FENGGWS030 | Heilongjiang, China |
| FENGGWS031 | Heilongjiang, China |
| FENGGWS032 | Heilongjiang, China |
| FENGGWS033 | Heilongjiang, China |
| FENGGWS034 | Heilongjiang, China |
| FENGGWS035 | Jilin, China |
| FENGGWS036 | Heilongjiang, China |
| FENGGWS037 | Heilongjiang, China |
| FENGGWS038 | Heilongjiang, China |
| FENGGWS039 | Heilongjiang, China |
| FENGGWS040 | Heilongjiang, China |
| FENGGWS041 | Heilongjiang, China |
| FENGGWS042 | Heilongjiang, China |
| FENGGWS043 | Jilin, China |
| FENGGWS044 | Heilongjiang, China |
| FENGGWS045 | Huanghuaihai region, China |
| FENGGWS046 | Xinan region, China |
| FENGGWS047 | Heilongjiang, China |
| FENGGWS048 | Heilongjiang, China |
| FENGGWS049 | Jilin, China |
| FENGGWS050 | Heilongjiang, China |
| FENGGWS051 | Liaoning, China |
| FENGGWS052 | Huanghuaihai region, China |
| FENGGWS053 | Heilongjiang, China |
| FENGGWS054 | Heilongjiang, China |
| FENGGWS055 | Heilongjiang, China |
| FENGGWS056 | Huanghuaihai region, China |
| FENGGWS057 | Jilin, China |
| FENGGWS058 | Jilin, China |
| FENGGWS059 | Jilin, China |
| FENGGWS061 | Jilin, China |
| FENGGWS062 | Jilin, China |
| FENGGWS063 | Jilin, China |
| FENGGWS064 | Jilin, China |
| FENGGWS065 | Jilin, China |
| FENGGWS066 | Jilin, China |
| FENGGWS067 | Jilin, China |
| FENGGWS068 | Jilin, China |
| FENGGWS069 | Jilin, China |
| FENGGWS070 | Jilin, China |
| FENGGWS071 | Jilin, China |
| FENGGWS072 | Jilin, China |
| FENGGWS073 | Jilin, China |
| FENGGWS074 | Jilin, China |
| FENGGWS075 | Xinan region, China |
| FENGGWS076 | Huanghuaihai region, China |
| FENGGWS077 | Jilin, China |
| FENGGWS078 | Liaoning, China |
| FENGGWS079 | Liaoning, China |
| FENGGWS080 | Jilin, China |
| FENGGWS081 | Heilongjiang, China |
| FENGGWS082 | Jilin, China |
| FENGGWS083 | Jilin, China |
| FENGGWS084 | Heilongjiang, China |
| FENGGWS085 | Heilongjiang, China |
| FENGGWS086 | Heilongjiang, China |
| FENGGWS087 | Jilin, China |
| FENGGWS088 | Jilin, China |
| FENGGWS089 | Jilin, China |
| FENGGWS090 | Jilin, China |
| FENGGWS091 | Jilin, China |
| FENGGWS092 | Jilin, China |
| FENGGWS093 | Jilin, China |
| FENGGWS094 | Jilin, China |
| FENGGWS095 | Jilin, China |
| FENGGWS096 | Jilin, China |
| FENGGWS097 | Jilin, China |
| FENGGWS098 | Jilin, China |
| FENGGWS099 | Jilin, China |
| FENGGWS100 | Jilin, China |
| FENGGWS101 | Jilin, China |
| FENGGWS102 | Jilin, China |
| FENGGWS103 | Liaoning, China |
| FENGGWS104 | Liaoning, China |
| FENGGWS105 | Jilin, China |
| FENGGWS106 | Jilin, China |
| FENGGWS107 | Jilin, China |
| FENGGWS108 | Huanghuaihai region, China |
| FENGGWS109 | Jilin, China |
| FENGGWS110 | Jilin, China |
| FENGGWS111 | Jilin, China |
| FENGGWS112 | Liaoning, China |
| FENGGWS113 | Jilin, China |
| FENGGWS114 | Heilongjiang, China |
| FENGGWS115 | Liaoning, China |
| FENGGWS116 | Heilongjiang, China |
| FENGGWS117 | Huanghuaihai region, China |
| FENGGWS118 | Jilin, China |
| FENGGWS119 | Jilin, China |
| FENGGWS120 | Jilin, China |
| FENGGWS121 | Jilin, China |
| FENGGWS122 | Liaoning, China |
| FENGGWS123 | Huanghuaihai region, China |
| FENGGWS124 | Jilin, China |
| FENGGWS125 | Xinan region, China |
| FENGGWS126 | Jilin, China |
| FENGGWS127 | Jilin, China |
| FENGGWS128 | Xinan region, China |
| FENGGWS129 | Jilin, China |
| FENGGWS130 | Jilin, China |
| FENGGWS131 | Other region of China |
| FENGGWS132 | Heilongjiang, China |
| FENGGWS133 | Xinan region, China |
| FENGGWS134 | Huanghuaihai region, China |
| FENGGWS135 | Jilin, China |
| FENGGWS136 | Jilin, China |
| FENGGWS138 | Liaoning, China |
| FENGGWS139 | Liaoning, China |
| FENGGWS140 | Liaoning, China |
| FENGGWS141 | Liaoning, China |
| FENGGWS142 | Liaoning, China |
| FENGGWS143 | Liaoning, China |
| FENGGWS144 | Liaoning, China |
| FENGGWS146 | Liaoning, China |
| FENGGWS147 | Liaoning, China |
| FENGGWS148 | Liaoning, China |
| FENGGWS149 | Heilongjiang, China |
| FENGGWS150 | Jilin, China |
| FENGGWS151 | Huanghuaihai region, China |
| FENGGWS152 | Other region of China |
| FENGGWS153 | Jilin, China |
| FENGGWS154 | Xinan region, China |
| FENGGWS155 | Liaoning, China |
| FENGGWS156 | Other region of China |
| FENGGWS157 | Jilin, China |
| FENGGWS158 | Xinan region, China |
| FENGGWS159 | Jilin, China |
| FENGGWS160 | Liaoning, China |
| FENGGWS161 | Other region of China |
| FENGGWS162 | Other region of China |
| FENGGWS163 | Jilin, China |
| FENGGWS164 | Liaoning, China |
| FENGGWS165 | Huanghuaihai region, China |
| FENGGWS166 | Liaoning, China |
| FENGGWS167 | Liaoning, China |
| FENGGWS168 | Liaoning, China |
| FENGGWS169 | Liaoning, China |
| FENGGWS170 | Liaoning, China |
| FENGGWS171 | Liaoning, China |
| FENGGWS172 | Other region of China |
| FENGGWS173 | Huanghuaihai region, China |
| FENGGWS174 | Liaoning, China |
| FENGGWS175 | Huanghuaihai region, China |
| FENGGWS176 | Jilin, China |
| FENGGWS177 | Liaoning, China |
| FENGGWS178 | Huanghuaihai region, China |
| FENGGWS179 | Huanghuaihai region, China |
| FENGGWS180 | Liaoning, China |
| FENGGWS181 | Jilin, China |
| FENGGWS182 | Jilin, China |
| FENGGWS183 | Liaoning, China |
| FENGGWS184 | Liaoning, China |
| FENGGWS185 | Liaoning, China |
| FENGGWS186 | Liaoning, China |
| FENGGWS187 | Huanghuaihai region, China |
| FENGGWS188 | Jilin, China |
| FENGGWS189 | Heilongjiang, China |
| FENGGWS191 | Heilongjiang, China |
| FENGGWS193 | Heilongjiang, China |
| FENGGWS194 | Heilongjiang, China |
| FENGGWS195 | Heilongjiang, China |
| FENGGWS196 | Heilongjiang, China |
| FENGGWS197 | Heilongjiang, China |
| FENGGWS198 | Heilongjiang, China |
| FENGGWS199 | Heilongjiang, China |
| FENGGWS200 | Heilongjiang, China |
| FENGGWS201 | Other region of China |
| FENGGWS202 | Heilongjiang, China |
| FENGGWS203 | Heilongjiang, China |
| FENGGWS204 | Jilin, China |
| FENGGWS205 | Heilongjiang, China |
| FENGGWS206 | Jilin, China |
| FENGGWS207 | Heilongjiang, China |
| FENGGWS208 | Huanghuaihai region, China |
| FENGGWS209 | Heilongjiang, China |
| FENGGWS210 | Heilongjiang, China |
| FENGGWS211 | Liaoning, China |
| FENGGWS212 | Jilin, China |
| FENGGWS213 | Heilongjiang, China |
| FENGGWS214 | Heilongjiang, China |
| FENGGWS215 | Heilongjiang, China |
| FENGGWS216 | Heilongjiang, China |
| FENGGWS217 | Jilin, China |
| FENGGWS218 | Jilin, China |
| FENGGWS219 | Heilongjiang, China |
| FENGGWS220 | Liaoning, China |
| FENGGWS221 | Heilongjiang, China |
| FENGGWS222 | Xinan region, China |
| FENGGWS223 | Jilin, China |
| FENGGWS224 | Heilongjiang, China |
| FENGGWS225 | Liaoning, China |
| FENGGWS226 | Heilongjiang, China |
| FENGGWS227 | Jilin, China |
| FENGGWS228 | Huanghuaihai region, China |
| FENGGWS229 | Heilongjiang, China |
| FENGGWS230 | Liaoning, China |
| FENGGWS231 | Huanghuaihai region, China |
| FENGGWS232 | Heilongjiang, China |
| FENGGWS233 | Heilongjiang, China |
| FENGGWS234 | Heilongjiang, China |
| FENGGWS235 | Jilin, China |
| FENGGWS236 | Huanghuaihai region, China |
| FENGGWS237 | Jilin, China |
| FENGGWS238 | Jilin, China |
| FENGGWS239 | Jilin, China |
| FENGGWS240 | Jilin, China |
| FENGGWS241 | Jilin, China |
| FENGGWS242 | Jilin, China |
| FENGGWS243 | Jilin, China |
| FENGGWS244 | Jilin, China |
| FENGGWS245 | Jilin, China |
| FENGGWS246 | Jilin, China |
| FENGGWS247 | Jilin, China |
| FENGGWS248 | Jilin, China |
| FENGGWS249 | Jilin, China |
| FENGGWS250 | Jilin, China |
| FENGGWS251 | Jilin, China |
| FENGGWS252 | Heilongjiang, China |
| FENGGWS253 | Jilin, China |
| FENGGWS254 | Jilin, China |
| FENGGWS255 | Jilin, China |
| FENGGWS256 | Jilin, China |
| FENGGWS257 | Xinan region, China |
| FENGGWS258 | Jilin, China |
| FENGGWS259 | Jilin, China |
| FENGGWS260 | Jilin, China |
| FENGGWS261 | Jilin, China |
| FENGGWS262 | Jilin, China |
| FENGGWS263 | Jilin, China |
| FENGGWS264 | Jilin, China |
| FENGGWS265 | Jilin, China |
| FENGGWS266 | Jilin, China |
| FENGGWS267 | Jilin, China |
| FENGGWS268 | Jilin, China |
| FENGGWS269 | Jilin, China |
| FENGGWS270 | Jilin, China |
| FENGGWS271 | Jilin, China |
| FENGGWS272 | Jilin, China |
| FENGGWS273 | Jilin, China |
| FENGGWS274 | Jilin, China |
| FENGGWS275 | Jilin, China |
| FENGGWS276 | Jilin, China |
| FENGGWS277 | Jilin, China |
| FENGGWS278 | Jilin, China |
| FENGGWS279 | Jilin, China |
| FENGGWS280 | Jilin, China |
| FENGGWS281 | Jilin, China |
| FENGGWS282 | Jilin, China |
| FENGGWS283 | Jilin, China |
| FENGGWS284 | Jilin, China |
| FENGGWS285 | Jilin, China |
| FENGGWS286 | Jilin, China |
| FENGGWS287 | Jilin, China |
| FENGGWS288 | Jilin, China |
| FENGGWS289 | Jilin, China |
| FENGGWS290 | Jilin, China |
| FENGGWS291 | Jilin, China |
| FENGGWS292 | Jilin, China |
| FENGGWS293 | Jilin, China |
| FENGGWS294 | Jilin, China |
| FENGGWS295 | Jilin, China |
| FENGGWS296 | Jilin, China |
| FENGGWS297 | Jilin, China |
| FENGGWS298 | Jilin, China |
| FENGGWS299 | Jilin, China |
| FENGGWS300 | Jilin, China |
| FENGGWS301 | Liaoning, China |
| FENGGWS302 | Liaoning, China |
| FENGGWS303 | Liaoning, China |
| FENGGWS304 | Liaoning, China |
| FENGGWS305 | Liaoning, China |
| FENGGWS306 | Liaoning, China |
| FENGGWS307 | Huanghuaihai region, China |
| FENGGWS308 | Heilongjiang, China |
| FENGGWS309 | Xinan region, China |
| FENGGWS310 | Huanghuaihai region, China |
| FENGGWS311 | Heilongjiang, China |
| FENGGWS312 | Heilongjiang, China |
| FENGGWS313 | Heilongjiang, China |
| FENGGWS314 | Heilongjiang, China |
| FENGGWS315 | Heilongjiang, China |
| FENGGWS316 | Heilongjiang, China |
| FENGGWS317 | Heilongjiang, China |
| FENGGWS318 | Heilongjiang, China |
| FENGGWS319 | Heilongjiang, China |
| FENGGWS320 | Heilongjiang, China |
| FENGGWS321 | Heilongjiang, China |
| FENGGWS322 | Heilongjiang, China |
| FENGGWS323 | Heilongjiang, China |
| FENGGWS324 | Jilin, China |
| FENGGWS325 | Jilin, China |
| FENGGWS326 | Jilin, China |
| FENGGWS327 | Jilin, China |
| FENGGWS328 | Jilin, China |
| FENGGWS329 | Jilin, China |
| FENGGWS330 | Jilin, China |
| FENGGWS332 | Jilin, China |
| FENGGWS333 | Liaoning, China |
| FENGGWS334 | Liaoning, China |
| FENGGWS335 | Other region of China |
| FENGGWS336 | Other region of China |
| FENGGWS337 | Other region of China |
| FENGGWS339 | Liaoning, China |
| FENGGWS340 | Other region of China |
| FENGGWS341 | Heilongjiang, China |
| FENGGWS342 | Heilongjiang, China |
| FENGGWS343 | Heilongjiang, China |
| FENGGWS344 | Heilongjiang, China |
| FENGGWS345 | Heilongjiang, China |
| FENGGWS346 | Jilin, China |
| FENGGWS347 | Jilin, China |
| FENGGWS348 | Jilin, China |
| FENGGWS349 | Jilin, China |
| FENGGWS350 | Liaoning, China |

**Supplementary Table S2.** Phenotypic analysis of seed oil content (SOC) and seed protein content (SPC) of 340 soybean accessions evaluated in different individual environments plus combined environment (CE).

| **Trait** | **Env.** | **Mean** | **Min** | **Max** | **SD** | **CV(%)** | **Skew.** | **Kurt.** | ***V*_g_** | ***V*_ge_** | ***V*_e_** | ***H*** |
| --- | --- | --- | --- | --- | --- | --- | --- | --- | --- | --- | --- | --- |
| SOC | CC22^*^ | 19.41 | 13.59 | 23.14 | 1.62 | 8.36 | -0.68 | 1.15 | 1.8 | 0.57 | 0.54 | 0.86 |
|  | CC23 | 19.21 | 13.77 | 24.35 | 1.60 | 8.37 | -0.21 | 0.52 |  |  |  |  |
|  | CC24 | 20.00 | 14.35 | 23.75 | 1.59 | 7.96 | -0.62 | 0.93 |  |  |  |  |
|  | JT24 | 20.56 | 14.35 | 24.67 | 1.57 | 7.62 | -0.79 | 1.31 |  |  |  |  |
|  | CE | 19.80 | 14.26 | 23.67 | 1.41 | 7.11 | -0.76 | 1.37 |  |  |  |  |
| SPC | CC22 | 39.75 | 31.72 | 46.21 | 2.22 | 5.59 | -0.11 | 0.23 | 2.74 | 1.36 | 1.37 | 0.79 |
|  | CC23 | 42.23 | 34.57 | 50.83 | 2.31 | 5.48 | 0.02 | 0.50 |  |  |  |  |
|  | CC24 | 41.65 | 36.37 | 47.34 | 1.91 | 4.58 | -0.10 | 0.02 |  |  |  |  |
|  | JT24 | 39.95 | 34.39 | 46.55 | 2.12 | 5.30 | 0.10 | 0.08 |  |  |  |  |
|  | CE | 40.91 | 34.45 | 46.58 | 1.79 | 4.36 | -0.10 | 0.48 |  |  |  |  |

*CC22 (Changchun 2022), CC23 (Changchun 2023), CC24 (Changchun 2024), JT24 (Jiutai 2024) and CE (Combined Environment); SD (Standard Deviation); CV (Coefficient of Variation); *H* (Broad-sense heritability)

**Supplementary Table S3.** Distribution of the SNPs used for GWAS across soybean chromosomes.

| **Chromosome** | **Number of SNP** | **Length of Chromosome** | **Number of SNP per 1 Mb** | **Average distance between SNPs** |
| --- | --- | --- | --- | --- |
| Chr01 | 146188 | 56831624 | 2572.30 | 388.76 |
| Chr02 | 138511 | 48577505 | 2851.34 | 350.71 |
| Chr03 | 199872 | 45779781 | 4365.94 | 229.05 |
| Chr04 | 213573 | 52389146 | 4076.67 | 245.30 |
| Chr05 | 90455 | 42234498 | 2141.73 | 466.92 |
| Chr06 | 209065 | 51416486 | 4066.11 | 245.94 |
| Chr07 | 138313 | 44630646 | 3099.06 | 322.68 |
| Chr08 | 128321 | 47837940 | 2682.41 | 372.80 |
| Chr09 | 200167 | 50189764 | 3988.20 | 250.74 |
| Chr10 | 154539 | 51566898 | 2996.86 | 333.68 |
| Chr11 | 63224 | 34766867 | 1818.51 | 549.91 |
| Chr12 | 99277 | 40091314 | 2476.27 | 403.84 |
| Chr13 | 170630 | 45874162 | 3719.52 | 268.85 |
| Chr14 | 138068 | 49042192 | 2815.29 | 355.21 |
| Chr15 | 256000 | 51756343 | 4946.25 | 202.17 |
| Chr16 | 176883 | 37887014 | 4668.70 | 214.19 |
| Chr17 | 177967 | 41641366 | 4273.80 | 233.98 |
| Chr18 | 255419 | 58018742 | 4402.35 | 227.15 |
| Chr19 | 216304 | 50746916 | 4262.41 | 234.61 |
| Chr20 | 170596 | 47904181 | 3561.19 | 280.81 |
| Total | 3343372 | 949183385 | 3522.37 | 283.90 |

**Supplementary Table S4.** SNP markers and stable QTLs significantly associated with seed oil content (SOC) and seed protein content (SPC) across different environments and models.

| **Trait name** | **Significant SNPs** | **Chr** | **Pos** | **-log_10_*P*** | **PVE(%)** | **Effect** | **MAF** | **Environment** | **Model** | **Stable QTL** | **Related QTLs** |
| --- | --- | --- | --- | --- | --- | --- | --- | --- | --- | --- | --- |
| SPC | Chr01_11010539 | 1 | 11010539 | 10.20 | 13.79 | -1.25 | 0.06 | JT24, CE | GLM, FarmCPU, BLINK | *qSPC1* |  |
| SPC | Chr01_13190977 | 1 | 13190977 | 6.71 | 1.26 | 0.63 | 0.22 | CC23 | FarmCPU |  |  |
| SOC | Chr01_51224066 | 1 | 51224066 | 8.88 | 24.13 | 0.75 | 0.06 | CC24 | GLM, FarmCPU, BLINK | *qSOC1* |  |
| SOC | Chr01_51224154 | 1 | 51224154 | 7.82 | 12.91 | 0.76 | 0.06 | CC22 | GLM, FarmCPU, BLINK |  |  |
| SOC | Chr01_53141395 | 1 | 53141395 | 8.28 | 1.68 | -0.35 | 0.13 | CE | GLM, BLINK |  |  |
| SPC | Chr02_9166079 | 2 | 9166079 | 8.21 | 0.63 | 0.32 | 0.35 | CE | FarmCPU |  |  |
| SOC | Chr02_9943578 | 2 | 9943578 | 7.80 | 0.63 | -0.22 | 0.34 | CE | FarmCPU |  |  |
| SOC | Chr02_11832415 | 2 | 11832415 | 10.50 | 0.70 | -0.74 | 0.08 | CC22 | GLM, BLINK |  |  |
| SOC | Chr02_12242717 | 2 | 12242717 | 7.06 | 2.38 | 0.39 | 0.16 | CC23 | FarmCPU |  |  |
| SOC | Chr02_15930382 | 2 | 15930382 | 10.02 | 0.92 | 0.73 | 0.09 | CC23 | FarmCPU |  |  |
| SOC | Chr02_30741723 | 2 | 30741723 | 6.67 | 0.83 | 0.54 | 0.42 | CC24 | FarmCPU |  |  |
| SPC | Chr02_35216537 | 2 | 35216537 | 10.48 | 2.16 | 0.81 | 0.20 | CC23 | FarmCPU |  |  |
| SOC | Chr02_36506109 | 2 | 36506109 | 7.28 | 0.85 | 0.22 | 0.45 | CE | FarmCPU |  |  |
| SPC | Chr02_39822002 | 2 | 39822002 | 6.73 | 0.30 | -0.36 | 0.33 | CC23 | FarmCPU |  |  |
| SOC | Chr03_3646690 | 3 | 3646690 | 8.15 | 2.64 | -0.35 | 0.12 | CE | GLM, FarmCPU, BLINK |  |  |
| SOC | Chr03_4030710 | 3 | 4030710 | 7.29 | 1.41 | 0.67 | 0.08 | JT24 | GLM, BLINK |  |  |
| SPC | Chr03_4918015 | 3 | 4918015 | 8.09 | 3.78 | -0.96 | 0.15 | CC24 | GLM, BLINK |  |  |
| SPC | Chr03_7999855 | 3 | 7999855 | 10.34 | 0.70 | -0.45 | 0.24 | CC22 | FarmCPU |  |  |
| SOC | Chr03_8854032 | 3 | 8854032 | 7.65 | 69.24 | 6.10 | 0.50 | JT24 | MLM, CMLM, MLMM, super, FarmCPU |  |  |
| SPC | Chr03_10006840 | 3 | 10006840 | 8.87 | 1.64 | 0.40 | 0.25 | CE | FarmCPU |  |  |
| SPC | Chr03_20905480 | 3 | 20905480 | 7.87 | 2.77 | 1.14 | 0.05 | CC22 | FarmCPU |  |  |
| SPC | Chr03_42717676 | 3 | 42717676 | 8.94 | 2.65 | -0.73 | 0.21 | CC23 | GLM, BLINK |  |  |
| SOC | Chr04_21080189 | 4 | 21080189 | 7.92 | 3.25 | -0.74 | 0.40 | CC23 | GLM, BLINK |  |  |
| SOC | Chr04_31571875 | 4 | 31571875 | 7.94 | 2.54 | -0.59 | 0.06 | CE | FarmCPU |  |  |
| SOC | Chr04_50622355 | 4 | 50622355 | 6.71 | 7.03 | 0.43 | 0.10 | CC22 | GLM, FarmCPU, BLINK |  |  |
| SPC | Chr05_886867 | 5 | 886867 | 9.63 | 2.35 | 0.47 | 0.26 | CE | GLM, BLINK |  |  |
| SOC | Chr05_13061479 | 5 | 13061479 | 9.41 | 1.77 | -0.63 | 0.11 | CC24 | FarmCPU |  |  |
| SOC | Chr05_32931181 | 5 | 32931181 | 8.48 | 0.84 | -0.34 | 0.20 | CC24 | FarmCPU |  |  |
| SPC | Chr05_35310933 | 5 | 35310933 | 9.05 | 0.76 | 0.51 | 0.45 | JT24 | GLM, FarmCPU, BLINK |  |  |
| SPC | Chr05_39345874 | 5 | 39345874 | 6.61 | 0.84 | -0.30 | 0.14 | CE | FarmCPU |  |  |
| SPC | Chr06_898974 | 6 | 898974 | 7.13 | 3.96 | 0.56 | 0.18 | CC24 | GLM, BLINK |  |  |
| SPC | Chr06_5204955 | 6 | 5204955 | 13.32 | 2.44 | -0.53 | 0.38 | CE | GLM, BLINK | *qSOC_SPC6.1* | AX-157493193 (Zhang *et al.* 2025) |
| SOC | Chr06_5209844 | 6 | 5209844 | 6.87 | 1.22 | 0.25 | 0.38 | CC23 | FarmCPU |  |  |
| SOC | Chr06_5294262 | 6 | 5294262 | 6.54 | 3.27 | -0.48 | 0.32 | JT24 | GLM, MLM, CMLM, FarmCPU, BLINK |  |  |
| SOC | Chr06_5404455 | 6 | 5404455 | 6.57 | 0.11 | -0.53 | 0.31 | JT24 | MLM, CMLM |  |  |
| SOC | Chr06_5507309 | 6 | 5507309 | 7.26 | 0.99 | 0.34 | 0.11 | CE | GLM, BLINK |  |  |
| SPC | Chr06_6450422 | 6 | 6450422 | 7.44 | 3.41 | -0.66 | 0.11 | JT24 | FarmCPU |  |  |
| SOC | Chr06_6694143 | 6 | 6694143 | 6.71 | 4.08 | 0.46 | 0.07 | CC24 | FarmCPU | *qSOC_SPC6.2* | cqSeed oil-016 & qSeed protein-015 (Pathan *et al*. 2013) |
| SPC | Chr06_6762512 | 6 | 6762512 | 7.55 | 5.29 | -0.72 | 0.11 | CC24, CE | GLM, BLINK, FarmCPU |  |  |
| SOC | Chr06_14974234 | 6 | 14974234 | 7.80 | 1.56 | -0.33 | 0.08 | CE | FarmCPU |  |  |
| SOC | Chr06_17905813 | 6 | 17905813 | 7.45 | 2.33 | 0.59 | 0.12 | CC24 | GLM, BLINK |  |  |
| SOC | Chr06_18345813 | 6 | 18345813 | 11.19 | 12.56 | -1.09 | 0.07 | CC24 | FarmCPU |  |  |
| SOC | Chr06_18361704 | 6 | 18361704 | 7.91 | 4.29 | -0.73 | 0.07 | CE | GLM, FarmCPU, BLINK |  |  |
| SOC | Chr06_18523086 | 6 | 18523086 | 10.90 | 1.62 | -0.45 | 0.18 | JT24 | FarmCPU |  |  |
| SOC | Chr06_34582695 | 6 | 34582695 | 8.23 | 2.40 | -0.32 | 0.25 | CE | GLM, BLINK |  |  |
| SOC | Chr06_34721520 | 6 | 34721520 | 7.70 | 2.21 | -0.60 | 0.23 | CC23 | GLM, BLINK |  |  |
| SOC | Chr06_38820099 | 6 | 38820099 | 12.72 | 3.15 | 0.56 | 0.21 | CC24 | FarmCPU |  |  |
| SOC | Chr06_43541487 | 6 | 43541487 | 8.81 | 1.29 | -0.55 | 0.38 | JT24 | FarmCPU |  |  |
| SOC | Chr06_45378420 | 6 | 45378420 | 6.67 | 11.51 | -0.40 | 0.18 | CC24 | GLM, BLINK |  |  |
| SPC | Chr07_14951443 | 7 | 14951443 | 9.18 | 1.36 | -0.60 | 0.39 | CC22 | GLM, FarmCPU, BLINK |  |  |
| SOC | Chr08_704436 | 8 | 704436 | 7.81 | 2.17 | 0.53 | 0.06 | CC23 | FarmCPU |  |  |
| SOC | Chr08_7831540 | 8 | 7831540 | 8.16 | 8.00 | -0.62 | 0.08 | CC23 | GLM, FarmCPU, BLINK | *qSOC8.1* | Seed oil 11-g2 (Yao *et al.* 2020) |
| SOC | Chr08_7870355 | 8 | 7870355 | 6.57 | 1.31 | 0.90 | 0.07 | JT24 | MLM, CMLM |  |  |
| SOC | Chr08_8004012 | 8 | 8004012 | 6.99 | 0.51 | 1.05 | 0.06 | JT24 | MLM, CMLM | *qSOC8.2* | Seed oil 1-1 (Mansur *et al.* 1993) |
| SOC | Chr08_8007019 | 8 | 8007019 | 7.41 | 1.22 | -1.13 | 0.06 | JT24 | MLM, CMLM |  |  |
| SOC | Chr08_8009399 | 8 | 8009399 | 7.39 | 0.00 | 1.13 | 0.06 | JT24 | MLM, CMLM |  |  |
| SOC | Chr08_8621551 | 8 | 8621551 | 6.64 | 10.97 | -1.07 | 0.07 | CC24 | MLM, CMLM | *qSOC8.3* | Seed oil 43-1 (Mao *et al.* 2013) |
| SOC | Chr08_8623213 | 8 | 8623213 | 6.57 | 0.01 | -0.94 | 0.08 | CC24 | MLM, CMLM |  |  |
| SOC | Chr08_8634846 | 8 | 8634846 | 6.78 | 0.01 | -0.95 | 0.08 | CC24 | MLM, CMLM |  |  |
| SOC | Chr08_8940647 | 8 | 8940647 | 6.78 | 0.00 | -1.14 | 0.05 | CC22 | MLM, CMLM | *qSOC_SPC8* | Seed oil 8-g13 & Seed protein 34-5 (Zhang *et al.* 2018; Lu *et al.* 2013) |
| SOC | Chr08_8943973 | 8 | 8943973 | 7.99 | 0.00 | 1.27 | 0.05 | CC22 | MLM, CMLM |  |  |
| SOC | Chr08_8994913 | 8 | 8994913 | 7.43 | 0.00 | 2.48 | 0.48 | CC22 | MLM, CMLM |  |  |
| SOC | Chr08_9003203 | 8 | 9003203 | 7.56 | 0.00 | 1.24 | 0.05 | CC22 | MLM, CMLM |  |  |
| SOC | Chr08_9016984 | 8 | 9016984 | 8.43 | 0.00 | 1.31 | 0.05 | CC22 | MLM, CMLM |  |  |
| SOC | Chr08_9028829 | 8 | 9028829 | 7.70 | 0.56 | 1.22 | 0.05 | CC22 | MLM, CMLM |  |  |
| SOC | Chr08_9041075 | 8 | 9041075 | 6.80 | 0.00 | 1.16 | 0.05 | CC22 | MLM, CMLM |  |  |
| SOC | Chr08_9041378 | 8 | 9041378 | 7.28 | 0.00 | 1.12 | 0.06 | CC22 | MLM, CMLM |  |  |
| SOC | Chr08_9046843 | 8 | 9046843 | 9.95 | 0.49 | 1.28 | 0.06 | CC22 | MLM, CMLM |  |  |
| SOC | Chr08_9054741 | 8 | 9054741 | 7.65 | 37.07 | -1.34 | 0.05 | CC22, JT24, CE | MLM, CMLM |  |  |
| SPC | Chr08_9054741 | 8 | 9054741 | 6.72 | 57.03 | -2.04 | 0.05 | CC22, CE | GLM, MLM, CMLM, MLMM, super, FarmCPU, BLINK |  |  |
| SOC | Chr08_9056324 | 8 | 9056324 | 7.36 | 61.01 | -1.40 | 0.05 | CC22, JT24, CE | MLM, CMLM, MLMM, super |  |  |
| SOC | Chr08_9058669 | 8 | 9058669 | 7.10 | 2.36 | -0.96 | 0.08 | CC22 | MLM, CMLM |  |  |
| SOC | Chr08_9059267 | 8 | 9059267 | 6.96 | 0.00 | 1.06 | 0.06 | CC22 | MLM, CMLM |  |  |
| SOC | Chr08_9059928 | 8 | 9059928 | 7.23 | 0.00 | 1.16 | 0.06 | CC22 | MLM, CMLM |  |  |
| SOC | Chr08_9060541 | 8 | 9060541 | 6.89 | 0.00 | 1.16 | 0.05 | CC22 | MLM, CMLM |  |  |
| SOC | Chr08_9062590 | 8 | 9062590 | 8.01 | 0.00 | -1.13 | 0.06 | CC22 | MLM, CMLM |  |  |
| SOC | Chr08_9068987 | 8 | 9068987 | 7.74 | 0.34 | 1.20 | 0.05 | CC22 | MLM, CMLM |  |  |
| SOC | Chr08_9071236 | 8 | 9071236 | 7.13 | 0.00 | 1.35 | 0.05 | CC22, JT24, CE | MLM, CMLM |  |  |
| SPC | Chr08_9071236 | 8 | 9071236 | 6.64 | 0.00 | 1.77 | 0.05 | CC22 | MLM, CMLM |  |  |
| SOC | Chr08_9071263 | 8 | 9071263 | 7.21 | 73.15 | 1.33 | 0.05 | CC22, CC24, JT24, CE | MLM, CMLM, MLMM, super, FarmCPU, GLM, BLINK |  |  |
| SPC | Chr08_9071263 | 8 | 9071263 | 7.94 | 2.51 | 1.94 | 0.05 | CC22 | MLM, CMLM |  |  |
| SOC | Chr08_9076006 | 8 | 9076006 | 8.47 | 0.27 | 1.27 | 0.05 | CC22 | MLM, CMLM |  |  |
| SOC | Chr08_9079037 | 8 | 9079037 | 7.61 | 0.26 | -1.17 | 0.06 | CC22 | MLM, CMLM |  |  |
| SOC | Chr08_9094556 | 8 | 9094556 | 7.08 | 0.00 | -1.15 | 0.05 | CC22 | MLM, CMLM |  |  |
| SOC | Chr08_9150120 | 8 | 9150120 | 7.15 | 1.77 | -1.12 | 0.06 | CC22 | MLM, CMLM |  |  |
| SOC | Chr08_19515869 | 8 | 19515869 | 10.46 | 1.83 | -0.46 | 0.12 | CC22 | FarmCPU |  |  |
| SPC | Chr08_24373040 | 8 | 24373040 | 9.48 | 2.15 | -0.66 | 0.09 | CE | FarmCPU |  |  |
| SPC | Chr08_24761790 | 8 | 24761790 | 8.01 | 4.95 | 0.32 | 0.18 | CE | FarmCPU |  |  |
| SPC | Chr08_27297760 | 8 | 27297760 | 6.98 | 2.40 | -0.68 | 0.25 | CE | FarmCPU |  |  |
| SOC | Chr09_6929070 | 9 | 6929070 | 14.18 | 1.89 | 0.40 | 0.28 | JT24, CE | FarmCPU | *qSOC9* |  |
| SOC | Chr09_6936859 | 9 | 6936859 | 7.65 | 1.89 | -0.25 | 0.28 | CE | GLM, BLINK |  |  |
| SOC | Chr09_6938994 | 9 | 6938994 | 7.80 | 4.64 | 0.41 | 0.29 | CC23 | GLM, FarmCPU, BLINK |  |  |
| SPC | Chr09_40290532 | 9 | 40290532 | 7.60 | 0.84 | 0.38 | 0.46 | CC23 | FarmCPU |  |  |
| SPC | Chr09_49248469 | 9 | 49248469 | 7.80 | 2.01 | -0.45 | 0.17 | CE | GLM, BLINK |  |  |
| SPC | Chr10_3944746 | 10 | 3944746 | 7.08 | 0.67 | 0.46 | 0.47 | CC23 | GLM, BLINK |  |  |
| SOC | Chr10_13397605 | 10 | 13397605 | 8.26 | 0.41 | -0.45 | 0.32 | JT24 | FarmCPU |  |  |
| SOC | Chr10_44549596 | 10 | 44549596 | 6.63 | 0.00 | 0.78 | 0.07 | CC24 | MLMM, super | *qSOC10* | Seed oil 39-16 (Wang *et al.* 2014) |
| SOC | Chr10_44549639 | 10 | 44549639 | 6.55 | 0.00 | -0.77 | 0.07 | CC24 | MLMM, super |  |  |
| SOC | Chr10_44549924 | 10 | 44549924 | 6.88 | 0.00 | -0.86 | 0.06 | CC24 | MLMM, super |  |  |
| SOC | Chr10_44552286 | 10 | 44552286 | 6.68 | 0.00 | -0.79 | 0.06 | CC24 | MLMM, super |  |  |
| SOC | Chr10_44563750 | 10 | 44563750 | 6.65 | 0.82 | -0.83 | 0.06 | CC24 | MLMM, super |  |  |
| SOC | Chr10_44564216 | 10 | 44564216 | 6.55 | 1.27 | -0.78 | 0.07 | CC24 | MLMM, super |  |  |
| SOC | Chr10_44583073 | 10 | 44583073 | 6.92 | 0.00 | 0.84 | 0.06 | CC24 | MLMM, super |  |  |
| SOC | Chr10_44583176 | 10 | 44583176 | 6.52 | 0.00 | -0.80 | 0.06 | CC24 | MLMM, super |  |  |
| SOC | Chr10_44584365 | 10 | 44584365 | 7.25 | 2.45 | 0.83 | 0.07 | CC24, JT24 | MLMM, super |  |  |
| SOC | Chr10_44584422 | 10 | 44584422 | 6.71 | 0.80 | 0.70 | 0.09 | CC24, JT24 | MLMM, super, FarmCPU |  |  |
| SOC | Chr10_44648239 | 10 | 44648239 | 6.56 | 0.00 | 0.84 | 0.06 | CC24 | MLMM, super |  |  |
| SOC | Chr10_44723614 | 10 | 44723614 | 6.69 | 0.00 | 0.81 | 0.06 | CC24 | MLMM, super |  |  |
| SOC | Chr11_32026898 | 11 | 32026898 | 10.89 | 1.97 | 0.55 | 0.06 | JT24 | FarmCPU |  |  |
| SOC | Chr11_33602175 | 11 | 33602175 | 7.41 | 1.00 | 0.25 | 0.31 | CE | FarmCPU |  |  |
| SOC | Chr12_1164390 | 12 | 1164390 | 7.43 | 1.56 | 0.29 | 0.37 | CC22 | FarmCPU |  |  |
| SPC | Chr12_1515702 | 12 | 1515702 | 9.18 | 1.32 | 0.60 | 0.39 | CC23 | GLM, BLINK |  |  |
| SPC | Chr12_4867236 | 12 | 4867236 | 6.87 | 3.10 | 0.44 | 0.33 | CC24 | GLM, BLINK | *qSPC12* | Seed protein 28-3 (Liang *et al.* 2010) |
| SPC | Chr12_4970475 | 12 | 4970475 | 6.61 | 9.42 | -0.71 | 0.31 | CC24 | MLMM, super |  |  |
| SOC | Chr12_20761693 | 12 | 20761693 | 7.65 | 3.03 | 5.99 | 0.50 | JT24 | MLM, CMLM |  |  |
| SPC | Chr12_36879339 | 12 | 36879339 | 6.72 | 9.09 | -0.92 | 0.15 | CC24 | MLMM, super |  |  |
| SPC | Chr12_37018901 | 12 | 37018901 | 8.86 | 2.38 | 0.67 | 0.24 | CC22 | GLM, FarmCPU, BLINK |  |  |
| SPC | Chr12_39353832 | 12 | 39353832 | 9.87 | 6.57 | 0.89 | 0.08 | CC23 | FarmCPU |  |  |
| SPC | Chr13_1031009 | 13 | 1031009 | 9.59 | 3.89 | 0.82 | 0.13 | CC22 | GLM, FarmCPU, BLINK |  |  |
| SPC | Chr13_16100245 | 13 | 16100245 | 6.99 | 0.91 | 0.68 | 0.19 | CC23 | FarmCPU |  |  |
| SPC | Chr13_31388821 | 13 | 31388821 | 6.81 | 0.92 | -0.43 | 0.41 | JT24 | GLM, BLINK |  |  |
| SOC | Chr14_9085000 | 14 | 9085000 | 7.17 | 3.80 | -0.33 | 0.20 | JT24 | GLM, BLINK |  |  |
| SOC | Chr14_16519869 | 14 | 16519869 | 7.46 | 1.84 | 0.45 | 0.31 | CC23 | FarmCPU |  |  |
| SOC | Chr14_18593170 | 14 | 18593170 | 6.59 | 1.56 | -0.55 | 0.42 | CC24 | FarmCPU |  |  |
| SOC | Chr14_36397214 | 14 | 36397214 | 10.09 | 4.16 | -0.43 | 0.46 | JT24 | GLM, BLINK |  |  |
| SPC | Chr14_40765817 | 14 | 40765817 | 6.59 | 1.45 | 0.46 | 0.14 | JT24 | FarmCPU |  |  |
| SPC | Chr15_5051172 | 15 | 5051172 | 7.47 | 0.65 | 0.33 | 0.20 | CE | FarmCPU |  |  |
| SOC | Chr15_10147967 | 15 | 10147967 | 7.85 | 11.85 | -0.90 | 0.23 | CC22, CE | GLM, FarmCPU, BLINK | *qSOC15* | mqSeed Oil-014 (Qi *et al.* 2011) |
| SOC | Chr15_32179431 | 15 | 32179431 | 7.90 | 1.04 | 0.21 | 0.41 | CE | FarmCPU |  |  |
| SOC | Chr15_40720636 | 15 | 40720636 | 14.83 | 0.74 | 0.38 | 0.23 | JT24 | FarmCPU |  |  |
| SOC | Chr15_43715693 | 15 | 43715693 | 10.96 | 4.04 | -0.61 | 0.09 | JT24 | GLM, BLINK | *qSOC_SPC15.1* |  |
| SPC | Chr15_43740554 | 15 | 43740554 | 11.86 | 1.89 | 0.84 | 0.36 | CC23 | FarmCPU |  |  |
| SPC | Chr15_50474682 | 15 | 50474682 | 11.39 | 0.75 | -0.60 | 0.12 | CE | FarmCPU | *qSOC_SPC15.2* |  |
| SOC | Chr15_50988987 | 15 | 50988987 | 7.70 | 1.50 | -0.28 | 0.19 | CE | GLM, BLINK |  |  |
| SPC | Chr15_51084729 | 15 | 51084729 | 9.09 | 5.64 | 0.78 | 0.13 | JT24 | GLM, FarmCPU, BLINK |  |  |
| SPC | Chr16_10290817 | 16 | 10290817 | 12.34 | 1.10 | 0.86 | 0.46 | CC22 | FarmCPU |  |  |
| SPC | Chr16_27116715 | 16 | 27116715 | 7.26 | 0.94 | 0.62 | 0.12 | CE | FarmCPU |  |  |
| SOC | Chr16_31445438 | 16 | 31445438 | 9.17 | 0.41 | 0.43 | 0.08 | JT24 | FarmCPU |  |  |
| SPC | Chr17_13248210 | 17 | 13248210 | 11.68 | 6.99 | 1.08 | 0.07 | CC23 | FarmCPU |  |  |
| SPC | Chr17_14541741 | 17 | 14541741 | 8.05 | 3.30 | 0.82 | 0.08 | CC22 | FarmCPU |  |  |
| SPC | Chr17_35092820 | 17 | 35092820 | 7.32 | 0.40 | 0.44 | 0.49 | CC22 | FarmCPU |  |  |
| SOC | Chr17_38854870 | 17 | 38854870 | 7.65 | 10.07 | 5.99 | 0.50 | JT24 | MLM, CMLM |  |  |
| SPC | Chr17_39593674 | 17 | 39593674 | 11.88 | 5.29 | -0.93 | 0.12 | CC22 | GLM, FarmCPU, BLINK |  |  |
| SOC | Chr17_40548588 | 17 | 40548588 | 8.47 | 0.37 | 0.36 | 0.13 | JT24 | FarmCPU |  |  |
| SPC | Chr18_29822581 | 18 | 29822581 | 6.92 | 4.39 | -0.92 | 0.35 | CC23 | GLM, BLINK |  |  |
| SPC | Chr18_33996780 | 18 | 33996780 | 8.18 | 4.73 | 1.24 | 0.11 | CC23 | GLM, BLINK |  |  |
| SOC | Chr18_48959325 | 18 | 48959325 | 6.57 | 0.40 | 0.41 | 0.07 | JT24 | FarmCPU |  |  |
| SPC | Chr18_52483191 | 18 | 52483191 | 6.92 | 1.50 | 0.69 | 0.37 | JT24 | FarmCPU |  |  |
| SPC | Chr18_54349986 | 18 | 54349986 | 6.53 | 0.00 | 0.83 | 0.35 | CC23 | MLMM, super | *qSPC18* | Seed protein 36-25 (Mao *et al.* 2013) |
| SPC | Chr18_54368477 | 18 | 54368477 | 6.75 | 0.00 | 0.87 | 0.39 | CC23 | MLM, CMLM, MLMM, super |  |  |
| SPC | Chr18_54376853 | 18 | 54376853 | 6.82 | 4.06 | -0.88 | 0.35 | CC23 | GLM, MLMM, super, FarmCPU, BLINK |  |  |
| SPC | Chr18_54389017 | 18 | 54389017 | 6.68 | 3.06 | -0.85 | 0.37 | CC23 | MLM, CMLM, MLMM, super |  |  |
| SPC | Chr18_54408341 | 18 | 54408341 | 6.96 | 1.82 | 0.86 | 0.37 | CC23, JT24, CE | MLM, CMLM, MLMM, super, FarmCPU, GLM, BLINK |  |  |
| SOC | Chr18_57784503 | 18 | 57784503 | 6.67 | 0.00 | 0.71 | 0.10 | CC24 | MLM, CMLM | *qSOC18* | rs20739 (Zhao *et al.* 2024) |
| SOC | Chr18_57785103 | 18 | 57785103 | 6.72 | 4.30 | 0.69 | 0.11 | CC24 | MLM, CMLM |  |  |
| SOC | Chr19_16123 | 19 | 16123 | 8.74 | 0.82 | 0.40 | 0.10 | JT24 | FarmCPU |  |  |
| SOC | Chr19_23094867 | 19 | 23094867 | 7.29 | 2.27 | 0.70 | 0.12 | CC22 | GLM, FarmCPU, BLINK |  |  |
| SOC | Chr19_48063616 | 19 | 48063616 | 11.91 | 2.22 | -0.42 | 0.34 | CC23 | FarmCPU |  |  |
| SOC | Chr19_50111837 | 19 | 50111837 | 9.45 | 7.27 | 0.59 | 0.10 | CC23 | GLM, BLINK |  |  |
| SPC | Chr20_943268 | 20 | 943268 | 7.81 | 0.30 | -0.50 | 0.19 | CC22 | FarmCPU |  |  |
| SPC | Chr20_2894434 | 20 | 2894434 | 11.96 | 4.73 | -0.80 | 0.15 | CE | GLM, FarmCPU, BLINK | *qSPC20* | Seed protein 36-26 (Mao *et al.* 2013) |
| SPC | Chr20_2925719 | 20 | 2925719 | 11.42 | 3.69 | 0.70 | 0.21 | JT24 | FarmCPU |  |  |
| SPC | Chr20_5091138 | 20 | 5091138 | 8.72 | 1.01 | 0.75 | 0.18 | CE | FarmCPU |  |  |
| SOC | Chr20_7962124 | 20 | 7962124 | 8.36 | 0.96 | -0.21 | 0.44 | CE | GLM, BLINK |  |  |

* Chr, Chromosome; Pos, position in the chromosome; PVE, phenotypic variance explained; MAF, minor allele frequency. Related QTLs represents the QTLs which overlap with the stable QTLs in this work.

**Supplementary Table S5.** Haplotype analysis of 15 haplotype blocks identified in the present study across the panel of 340 soybean accessions.

| **Block** | **Chr** | **Hap** | **Markers** | | | | | | **Freq** | **Group_SOC** | **Mean_SOC** | **Group_SPC** | **Mean_SPC** |
| --- | --- | --- | --- | --- | --- | --- | --- | --- | --- | --- | --- | --- | --- |
| Hap-1 | Chr01 | POS | 51224066 | 51224154 |  |  |  |  |  |  |  |  |  |
|  |  | ALLELE | C/A | G/A |  |  |  |  |  |  |  |  |  |
|  |  | Hap-1A | C | G |  |  |  |  | 314 | a | 19.95 | a | 40.93 |
|  |  | Hap-1B | A | A |  |  |  |  | 16 | b | 17.42 | b | 39.69 |
| Hap-6.1 | Chr06 | POS | 5204955 | 5209844 | 5294262 |  |  |  |  |  |  |  |  |
|  |  | ALLELE | A | T/G | C/T |  |  |  |  |  |  |  |  |
|  |  | Hap-6.1A | G | T | C |  |  |  | 160 | a | 20.16 | b | 40.55 |
|  |  | Hap-6.1B | A | G | T |  |  |  | 65 | b | 19.45 | a | 41.58 |
|  |  | Hap-6.1C | A | G | C |  |  |  | 24 | ab | 19.95 | ab | 41.10 |
|  |  | Hap-6.1D | G | T | T |  |  |  | 7 | c | 17.65 | c | 39.03 |
| Hap-6.2 | Chr06 | POS | 6694143 | 6762512 |  |  |  |  |  |  |  |  |  |
|  |  | ALLELE | A/G | A/G |  |  |  |  |  |  |  |  |  |
|  |  | Hap-6.2A | A | A |  |  |  |  | 285 | b | 19.76 | a | 41.16 |
|  |  | Hap-6.2B | G | G |  |  |  |  | 18 | a | 20.46 | b | 39.09 |
|  |  | Hap-6.2C | A | G |  |  |  |  | 4 | ab | 19.92 | ab | 39.90 |
| Hap-8.1 | Chr08 | POS | 7831540 | 7870355 |  |  |  |  |  |  |  |  |  |
|  |  | ALLELE | A/G | T/C |  |  |  |  |  |  |  |  |  |
|  |  | Hap-8.1A | A | T |  |  |  |  | 304 | a | 20.02 | a | 41.02 |
|  |  | Hap-8.1B | G | C |  |  |  |  | 21 | b | 17.40 | b | 39.02 |
|  |  | Hap-8.1C | G | T |  |  |  |  | 4 | b | 18.10 | a | 41.94 |
| Hap-8.2 | Chr08 | POS | 8004012 | 8007019 | 8009399 |  |  |  |  |  |  |  |  |
|  |  | ALLELE | T/C | G/T | T/C |  |  |  |  |  |  |  |  |
|  |  | Hap-8.2A | T | G | T |  |  |  | 312 | a | 19.99 | a | 41.01 |
|  |  | Hap-8.2B | C | T | C |  |  |  | 14 | b | 17.20 | b | 39.79 |
| Hap-8.3 | Chr08 | POS | 8621551 | 8623213 | 8634846 |  |  |  |  |  |  |  |  |
|  |  | ALLELE | A/G | A/G | C/T |  |  |  |  |  |  |  |  |
|  |  | Hap-8.3A | A | A | C |  |  |  | 303 | a | 20.00 | a | 41.04 |
|  |  | Hap-8.3B | G | G | T |  |  |  | 19 | b | 17.05 | b | 39.40 |
| Hap-8.4 | Chr08 | POS | 8940647 | 8943973 | 9003203 | 9016984 | 9028829 | 9041075 |  |  |  |  |  |
|  |  | ALLELE | C/T | G/A | G/A | G/A | G/A | G/A |  |  |  |  |  |
|  |  | Hap-8.4A | C | G | G | G | G | G | 263 | a | 20.03 | a | 40.97 |
|  |  | Hap-8.4B | T | A | A | A | A | A | 10 | b | 16.68 | b | 38.63 |
|  |  | POS | 9041378 | 9046843 | 9054741 | 9056324 | 9059267 | 9059267 |  |  |  |  |  |
|  |  | ALLELE | G/A | T/C | G/T | A/G | G/A | G/A |  |  |  |  |  |
|  |  | Hap-8.4A | G | T | G | A | G | G |  |  |  |  |  |
|  |  | Hap-8.4B | A | C | T | G | A | A |  |  |  |  |  |
|  |  | POS | 9059928 | 9060541 | 9062590 | 9068987 | 9071236 | 9071263 |  |  |  |  |  |
|  |  | ALLELE | G/A | G/A | C/T | G/A | G/A | G/A |  |  |  |  |  |
|  |  | Hap-8.4A | G | G | C | G | G | G |  |  |  |  |  |
|  |  | Hap-8.4B | A | A | T | A | A | A |  |  |  |  |  |
|  |  | POS | 9076006 | 9079037 | 9094556 | 9150120 |  |  |  |  |  |  |  |
|  |  | ALLELE | T/A | A/C | C/T | G/T |  |  |  |  |  |  |  |
|  |  | Hap-8.4A | T | A | C | G |  |  |  |  |  |  |  |
|  |  | Hap-8.4B | A | C | T | T |  |  |  |  |  |  |  |
| Hap-9 | Chr09 | POS | 6929070 | 6936859 | 6938994 |  |  |  |  |  |  |  |  |
|  |  | ALLELE | C/T | G/A | G/T |  |  |  |  |  |  |  |  |
|  |  | Hap-9A | C | G | G |  |  |  | 229 | b | 19.58 | a | 41.02 |
|  |  | Hap-9B | T | A | T |  |  |  | 79 | a | 20.37 | a | 40.70 |
| Hap-10 | Chr10 | POS | 44549596 | 44549639 | 44549924 | 44552286 | 44563750 | 44564216 |  |  |  |  |  |
|  |  | ALLELE | C/T | T/G | T/A | G/A | G/C | G/A |  |  |  |  |  |
|  |  | Hap-10A | T | G | A | A | C | A | 263 | a | 19.98 | a | 40.84 |
|  |  | Hap-10B | C | T | T | G | G | G | 12 | b | 18.39 | a | 41.68 |
|  |  | POS | 44583073 | 44583176 | 44584365 | 44584422 | 44648239 |  |  |  |  |  |  |
|  |  | ALLELE | G/T | T/C | A/G | C/T | C/T |  |  |  |  |  |  |
|  |  | Hap-10A | T | C | G | T | T |  |  |  |  |  |  |
|  |  | Hap-10B | G | T | A | C | C |  |  |  |  |  |  |
| Hap-12 | Chr12 | POS | 4867236 | 4970475 |  |  |  |  |  |  |  |  |  |
|  |  | ALLELE | G/C | C/G |  |  |  |  |  |  |  |  |  |
|  |  | Hap-12A | G | C |  |  |  |  | 219 | a | 19.79 | a | 41.87 |
|  |  | Hap-12B | C | G |  |  |  |  | 91 | a | 19.67 | b | 41.02 |
| Hap-15.1 | Chr15 | POS | 43715693 | 43740554 |  |  |  |  |  |  |  |  |  |
|  |  | ALLELE | T/C | G/C |  |  |  |  |  |  |  |  |  |
|  |  | Hap-15.1A | T | G |  |  |  |  | 124 | a | 20.04 | a | 41.05 |
|  |  | Hap-15.1B | T | C |  |  |  |  | 32 | b | 18.77 | a | 40.41 |
|  |  | Hap-15.1C | C | C |  |  |  |  | 6 | ab | 19.68 | a | 41.29 |
|  |  | Hap-15.1D | C | G |  |  |  |  | 4 | ab | 19.80 | a | 41.54 |
| Hap-15.2 | Chr15 | POS | 50988987 | 51084729 |  |  |  |  |  |  |  |  |  |
|  |  | ALLELE | G/A | T/C |  |  |  |  |  |  |  |  |  |
|  |  | Hap-15.2A | G | C |  |  |  |  | 224 | b | 19.49 | a | 41.05 |
|  |  | Hap-15.2B | A | C |  |  |  |  | 59 | a | 21.15 | b | 39.90 |
|  |  | Hap-15.2C | G | T |  |  |  |  | 34 | b | 19.44 | a | 41.61 |
| Hap-18.1 | Chr18 | POS | 54349986 | 54389017 | 54408341 |  |  |  |  |  |  |  |  |
|  |  | ALLELE | T/C | C/A | C/T |  |  |  |  |  |  |  |  |
|  |  | Hap-18.1A | T | A | T |  |  |  | 180 | a | 19.89 | a | 41.39 |
|  |  | Hap-18.1B | C | C | C |  |  |  | 90 | a | 19.81 | b | 40.10 |
|  |  | Hap-18.1C | T | C | C |  |  |  | 6 | b | 18.17 | b | 39.46 |
| Hap-18.2 | Chr18 | POS | 57784503 | 57785103 |  |  |  |  |  |  |  |  |  |
|  |  | ALLELE | T/A | G/A |  |  |  |  |  |  |  |  |  |
|  |  | Hap-18.2A | T | G |  |  |  |  | 296 | a | 20.00 | a | 40.93 |
|  |  | Hap-18.2B | A | A |  |  |  |  | 29 | b | 18.26 | a | 40.72 |
| Hap-20 | Chr20 | POS | 2894434 | 2925719 |  |  |  |  |  |  |  |  |  |
|  |  | ALLELE | T/C | A/G |  |  |  |  |  |  |  |  |  |
|  |  | Hap-20A | T | A |  |  |  |  | 249 | a | 20.07 | c | 40.52 |
|  |  | Hap-20B | C | G |  |  |  |  | 32 | b | 18.87 | a | 42.69 |
|  |  | Hap-20C | T | G |  |  |  |  | 25 | b | 19.50 | b | 41.62 |
|  |  | Hap-20D | C | A |  |  |  |  | 6 | b | 18.61 | ab | 41.86 |

**Supplementary Table S6.** Phenotypic variance explained (PVE) by the identified 17 stable QTLs in the combined environment.

| **QTL** | **SNP/Haplotype block** | **PVE** | |
| --- | --- | --- | --- |
|  |  | **SOC** | **SPC** |
| *qSOC1* | Chr01_11010539 | 2.07 | 2.32 |
| *qSPC1* | Hap-1 | 0.59 | 2.36 |
| *qSOC_SPC6.1* | Hap-6.1 | 8.78 | 7.50 |
| *qSOC_SPC6.2* | Hap-6.2 | 1.28 | 2.88 |
| *qSOC8.1* | Hap-8.1 | 3.17 | 0.66 |
| *qSOC8.2* | Hap-8.2 | 2.66 | 2.90 |
| *qSOC8.3* | Hap-8.3 | 0.75 | 2.23 |
| *qSOC_SPC8* | Hap-8.4 | 9.25 | 5.69 |
| *qSOC9* | Hap-9 | 3.09 | 2.33 |
| *qSOC10* | Hap-10 | 9.92 | 9.12 |
| *qSPC12* | Hap-12 | 2.40 | 1.32 |
| *qSOC15* | Chr15_10147967 | 0.55 | 0.48 |
| *qSOC_SPC15.1* | Hap-15.1 | 1.98 | 2.55 |
| *qSOC_SPC15.2* | Hap-15.2 | 4.89 | 3.64 |
| *qSPC18* | Hap-18.1 | 4.72 | 9.51 |
| *qSOC18* | Hap-18.2 | 3.30 | 1.57 |
| *qSPC20* | Hap-20 | 8.79 | 11.87 |
| (Residuals) | - | 31.80 | 31.08 |

**Supplementary Table S7.** Putative candidate genes selected based on the *in-silico* analysis and gene function annotation across the 17 QTLs.

| **Gene_ID** | **Annotations** |
| --- | --- |
| *Glyma.01G067200* | lipid metabolic process |
| *Glyma.01G067300* | protein desumoylation, vegetative to reproductive phase transition of meristem |
| *Glyma.01G067600* | monosaccharide transport |
| *Glyma.01G175000* | cellular process involved in reproduction; protein autophosphorylation; protein phosphorylation |
| *Glyma.01G175100* | post-translational protein modification |
| *Glyma.01G175500* | sterol biosynthetic process |
| *Glyma.01G176000* | embryo development ending in seed dormancy; intracellular protein transport; protein N-linked glycosylation; protein glycosylation; protein targeting to vacuole |
| *Glyma.01G176300* | embryo development ending in seed dormancy; embryo sac cellularization; endosperm development; intracellular protein transport |
| *Glyma.06G067300* | protein phosphorylation |
| *Glyma.06G067700* | response to far red light; response to red light; protein dimerization activity |
| *Glyma.06G068100* | protein autophosphorylation; protein phosphorylation |
| *Glyma.06G068300* | maltose metabolic process; starch biosynthetic process |
| *Glyma.06G068400* | ubiquitin-dependent protein catabolic process |
| *Glyma.06G068600* | fatty acid metabolic process; gluconeogenesis; glycolysis |
| *Glyma.06G068700* | fatty acid metabolic process; gluconeogenesis; glycolysis |
| *Glyma.06G069000* | galactolipid biosynthetic process |
| *Glyma.06G069100* | photosynthetic electron transport in photosystem I; photosystem II assembly |
| *Glyma.06G069500* | ER to Golgi vesicle-mediated transport; amino acid import; basic amino acid transport; embryo development ending in seed dormancy; protein targeting to membrane; transmembrane transport; transport |
| *Glyma.06G069600* | glycolysis; water transport |
| *Glyma.06G085900* | aromatic amino acid family biosynthetic process |
| *Glyma.06G086600* | embryo development ending in seed dormancy; embryonic pattern specification; meristem initiation; organ morphogenesis; seed maturation |
| *Glyma.06G086700* | fatty acid biosynthetic process; long-chain fatty acid metabolic process |
| *Glyma.06G086800* | protein phosphorylation |
| *Glyma.06G087500* | protein phosphorylation |
| *Glyma.06G087800* | N-terminal protein myristoylation; proteasomal protein catabolic process; protein homooligomerization |
| *Glyma.06G087900* | N-terminal protein myristoylation; proteolysis |
| *Glyma.06G088000* | N-terminal protein myristoylation; embryo development ending in seed dormancy; ovule development |
| *Glyma.06G088200* | amino acid transport |
| *Glyma.08G101100* | SRP-dependent cotranslational protein targeting to membrane |
| *Glyma.08G101500* | transmembrane transport; transport |
| *Glyma.08G101700* | transmembrane transport; transport |
| *Glyma.08G101900* | N-terminal protein myristoylation; amino acid import; amino acid transport; basic amino acid transport; endoplasmic reticulum unfolded protein response; protein targeting to membrane |
| *Glyma.08G102100* | fatty acid biosynthetic process; very long-chain fatty acid metabolic process |
| *Glyma.08G102400* | galactolipid biosynthetic process; negative regulation of transcription |
| *Glyma.08G102600* | protein phosphorylation; tissue development |
| *Glyma.08G102700* | organ morphogenesis; protein desumoylation; tissue development; vegetative to reproductive phase transition of meristem |
| *Glyma.08G102900* | lipid oxidation |
| *Glyma.08G103000* | nitrogen compound metabolic process |
| *Glyma.08G103100* | protein ubiquitination |
| *Glyma.08G103200* | oligosaccharide metabolic process |
| *Glyma.08G103300* | oproteolysis |
| *Glyma.08G103600* | signal peptide processing |
| *Glyma.08G104100* | floral organ development; leaf formation; organ growth |
| *Glyma.08G104300* | photorespiration; proteasome core complex assembly; response to misfolded protein; ubiquitin-dependent protein catabolic process |
| *Glyma.08G104600* | oligopeptide transport |
| *Glyma.08G105000* | cellular response to sucrose stimulus; protein import into peroxisome matrix |
| *Glyma.08G111700* | gluconeogenesis; methionine biosynthetic process; proteasomal protein catabolic process; protein sumoylation |
| *Glyma.08G111800* | gluconeogenesis; methionine biosynthetic process; proteasomal protein catabolic process; protein sumoylation |
| *Glyma.08G112000* | protein glycosylation; protein targeting to vacuole |
| *Glyma.08G112300* | lipid transport |
| *Glyma.08G112600* | amino acid transport |
| *Glyma.08G113200* | hydrolase activity; protein serine/threonine phosphatase activity |
| *Glyma.08G113400* | polyamine transport |
| *Glyma.08G115800* | cysteine biosynthetic process; glycolysis; regulation of protein localization |
| *Glyma.08G116300* | proteolysis |
| *Glyma.08G116400* | proteolysis |
| *Glyma.08G116500* | protein phosphorylation |
| *Glyma.08G116600* | protein desumoylation; protein phosphorylation |
| *Glyma.08G116900* | proteolysis |
| *Glyma.08G117000* | embryo development ending in seed dormancy; protein phosphorylation |
| *Glyma.08G117100* | transmembrane transport; transport |
| *Glyma.08G117200* | glycolysis; protein autophosphorylation; protein phosphorylation |
| *Glyma.08G118200* | nitrate transport; regulation of transcription, DNA-dependent |
| *Glyma.08G118600* | double fertilization forming a zygote and endosperm |
| *Glyma.08G119200* | N-terminal protein myristoylation |
| *Glyma.08G119300* | protein retention in ER lumen; protein transport |
| *Glyma.08G119700* | protein ubiquitination |
| *Glyma.09G068000* | protein transport; vesicle-mediated transport |
| *Glyma.09G068600* | positive regulation of transcription, DNA-dependent; post-translational protein modification; protein deubiquitination; protein ubiquitination |
| *Glyma.09G068700* | embryo development ending in seed dormancy; leaf development; leaf morphogenesis; regulation of flower development; regulation of transcription, DNA-dependent; seed dormancy process |
| *Glyma.10G212200* | proteasomal ubiquitin-dependent protein catabolic process; ubiquitin-dependent protein catabolic process |
| *Glyma.10G212500* | proteolysis |
| *Glyma.10G212700* | endoplasmic reticulum unfolded protein response; protein targeting to membrane |
| *Glyma.10G213100* | glucose catabolic process |
| *Glyma.10G213200* | transmembrane transport; transport |
| *Glyma.10G213400* | photorespiration; proteasome core complex assembly; response to misfolded protein; ubiquitin-dependent protein catabolic process |
| *Glyma.12G065600* | protein transport |
| *Glyma.12G066200* | cellular modified amino acid biosynthetic process; glycolysis; transmembrane transport; transport |
| *Glyma.12G067700* | Golgi vesicle transport; N-terminal protein myristoylation |
| *Glyma.12G068100* | embryo sac egg cell differentiation |
| *Glyma.12G068500* | protein phosphorylation |
| *Glyma.15G127400* | aromatic amino acid family biosynthetic process; cysteine metabolic process |
| *Glyma.15G127500* | carbohydrate metabolic process |
| *Glyma.15G127600* | embryo development ending in seed dormancy |
| *Glyma.15G127700* | fatty acid beta-oxidation; nuclear-transcribed mRNA catabolic process |
| *Glyma.15G127800* | photosynthesis |
| *Glyma.15G128000* | L-phenylalanine catabolic process; cellular amino acid metabolic process |
| *Glyma.15G232200* | cellular response to light stimulus; cellular response to nitrogen starvation; |
| *Glyma.15G232300* | protein targeting to mitochondrion; translation |
| *Glyma.15G232900* | endosomal transport; nuclear-transcribed mRNA catabolic process; protein ubiquitination |
| *Glyma.15G233000* | endosomal transport; nuclear-transcribed mRNA catabolic process; protein ubiquitination |
| *Glyma.15G272300* | glucose catabolic process; protein autoubiquitination; protein ubiquitination |
| *Glyma.15G273300* | photosynthesis |
| *Glyma.15G273600* | ER to Golgi vesicle-mediated transport; amino acid import; amino acid transport; proteasomal protein catabolic process; protein targeting to membrane; transmembrane transport; transport |
| *Glyma.15G273900* | photorespiration; photosynthesis; proteasome core complex assembly; response to misfolded protein; ubiquitin-dependent protein catabolic process |
| *Glyma.18G257700* | carbohydrate metabolic process; galactose metabolic process; hexose metabolic process |
| *Glyma.18G257800* | protein phosphorylation |
| *Glyma.18G258000* | transferase activity; transferase activity, transferring acyl groups other than amino-acyl groups |
| *Glyma.18G258100* | diacylglycerol O-acyltransferase activity |
| *Glyma.18G258300* | oligopeptide transport; protein phosphorylation |
| *Glyma.18G258500* | N-terminal protein myristoylation |
| *Glyma.18G258800* | protein transport |
| *Glyma.18G259100* | very long-chain fatty acid biosynthetic process |
| *Glyma.18G299400* | vesicle-mediated transport |
| *Glyma.18G299600* | Phosphoenolpyruvate carboxylase family protein |
| *Glyma.18G299700* | Phosphoenolpyruvate carboxylase family protein |
| *Glyma.18G299900* | methyltransferase activity |
| *Glyma.18G300100* | protein N-linked glycosylation; protein phosphorylation |
| *Glyma.18G300200* | (1->3)-beta-D-glucan biosynthetic process; callose deposition in cell wall; cytokinesis by cell plate formation; methylation-dependent chromatin silencing; microsporogenesis; microtubule cytoskeleton organization |
| *Glyma.18G300300* | cellular process involved in reproduction; protein ubiquitination |
| *Glyma.18G300400* | oligopeptide transport |
| *Glyma.18G300600* | protein transport |
| *Glyma.18G300700* | proteolysis |
| *Glyma.20G025900* | protein domain specific binding; protein phosphorylated amino acid binding |
| *Glyma.20G026100* | cotyledon development; embryo development ending in seed dormancy; fatty acid beta-oxidation; proteasomal ubiquitin-dependent protein catabolic process; proteasome assembly; proteasome core complex assembly; protein catabolic process; response to misfolded protein; ubiquitin-dependent protein catabolic process |
| *Glyma.20G026200* | regulation of carbohydrate metabolic process |
| *Glyma.20G026300* | carbohydrate metabolic process |
| *Glyma.20G026400* | response to fructose stimulus; response to glucose stimulus; response to sucrose stimulus |
| *Glyma.20G026700* | carbohydrate metabolic process; starch biosynthetic process |
| *Glyma.20G026800* | regulation of carbohydrate metabolic process |
| *Glyma.20G026900* | embryo development ending in seed dormancy; ovule development; photosynthesis; positive regulation of transcription, DNA-dependent; regulation of protein dephosphorylation; transcription from plastid promoter |

**Supplementary Table S8.** Variant annotation of selected putative candidate genes (only 104 among the total 121 genes selected based on the in-silico analysis and gene function annotation showed variant annotation).

| **Gene** | **Annotation** | **Putative impact** |
| --- | --- | --- |
| *Glyma.01G175100* | upstream_gene_variant,intron_variant,missense_variant,3_prime_UTR_variant,downstream_gene_variant | MODERATE,MODIFIER |
| *Glyma.01G175500* | upstream_gene_variant,intron_variant,3_prime_UTR_variant,downstream_gene_variant | MODIFIER |
| *Glyma.01G176000* | upstream_gene_variant,synonymous_variant,missense_variant,intron_variant,downstream_gene_variant | MODERATE,LOW,MODIFIER |
| *Glyma.06G067300* | downstream_gene_variant,splice_donor_variant&intron_variant,missense_variant,intron_variant,5_prime_UTR_variant,upstream_gene_variant | HIGH,MODERATE,MODIFIER |
| *Glyma.06G067700* | upstream_gene_variant,5_prime_UTR_premature_start_codon_gain_variant,5_prime_UTR_variant,intron_variant,3_prime_UTR_variant,downstream_gene_variant | LOW,MODIFIER |
| *Glyma.06G068100* | upstream_gene_variant,missense_variant,downstream_gene_variant | MODERATE,MODIFIER |
| *Glyma.06G068300* | upstream_gene_variant,5_prime_UTR_variant,intron_variant,missense_variant,splice_region_variant&intron_variant,downstream_gene_variant | MODERATE,LOW,MODIFIER |
| *Glyma.06G068600* | downstream_gene_variant,intron_variant,synonymous_variant,upstream_gene_variant | LOW,MODIFIER |
| *Glyma.06G068700* | downstream_gene_variant,synonymous_variant,missense_variant,intron_variant,upstream_gene_variant | MODERATE,LOW,MODIFIER |
| *Glyma.06G069000* | upstream_gene_variant,intron_variant,synonymous_variant,downstream_gene_variant | LOW,MODIFIER |
| *Glyma.06G069100* | downstream_gene_variant,intron_variant,5_prime_UTR_variant,upstream_gene_variant | MODIFIER |
| *Glyma.06G069500* | upstream_gene_variant,missense_variant,intron_variant,synonymous_variant,downstream_gene_variant | MODERATE,LOW,MODIFIER |
| *Glyma.06G069600* | downstream_gene_variant,synonymous_variant,intron_variant,missense_variant,upstream_gene_variant | MODERATE,LOW,MODIFIER |
| *Glyma.06G085900* | downstream_gene_variant,3_prime_UTR_variant,intron_variant,5_prime_UTR_variant,upstream_gene_variant | MODIFIER |
| *Glyma.06G086600* | upstream_gene_variant,5_prime_UTR_variant,5_prime_UTR_premature_start_codon_gain_variant,intron_variant,splice_region_variant&intron_variant,synonymous_variant,stop_gained,downstream_gene_variant | HIGH,LOW,MODIFIER |
| *Glyma.06G086700* | upstream_gene_variant,intron_variant,missense_variant,synonymous_variant,downstream_gene_variant | MODERATE,LOW,MODIFIER |
| *Glyma.06G086800* | downstream_gene_variant,intron_variant,3_prime_UTR_variant,synonymous_variant,splice_acceptor_variant&intron_variant,missense_variant,upstream_gene_variant | HIGH,MODERATE,LOW,MODIFIER |
| *Glyma.06G087500* | upstream_gene_variant,5_prime_UTR_variant,downstream_gene_variant | MODIFIER |
| *Glyma.06G087800* | upstream_gene_variant,splice_region_variant&intron_variant,5_prime_UTR_variant,intron_variant,synonymous_variant,splice_region_variant&synonymous_variant,3_prime_UTR_variant,downstream_gene_variant | LOW,MODIFIER |
| *Glyma.06G087900* | downstream_gene_variant,3_prime_UTR_variant,intron_variant,splice_region_variant&intron_variant,5_prime_UTR_variant,missense_variant,5_prime_UTR_premature_start_codon_gain_variant,upstream_gene_variant | MODERATE,LOW,MODIFIER |
| *Glyma.06G088000* | upstream_gene_variant,missense_variant,intron_variant,5_prime_UTR_variant,synonymous_variant,downstream_gene_variant,3_prime_UTR_variant | MODERATE,LOW,MODIFIER |
| *Glyma.06G088200* | upstream_gene_variant,5_prime_UTR_variant,splice_region_variant&synonymous_variant,intron_variant,synonymous_variant,splice_region_variant&intron_variant,missense_variant,3_prime_UTR_variant,downstream_gene_variant | MODERATE,LOW,MODIFIER |
| *Glyma.08G101100* | upstream_gene_variant,intron_variant,synonymous_variant,3_prime_UTR_variant,downstream_gene_variant | LOW,MODIFIER |
| *Glyma.08G101500* | upstream_gene_variant,synonymous_variant,splice_region_variant&intron_variant,intron_variant,missense_variant,downstream_gene_variant | MODERATE,LOW,MODIFIER |
| *Glyma.08G101700* | downstream_gene_variant,intron_variant,5_prime_UTR_variant,upstream_gene_variant | MODIFIER |
| *Glyma.08G101900* | upstream_gene_variant,intron_variant,downstream_gene_variant | MODIFIER |
| *Glyma.08G102100* | downstream_gene_variant,intron_variant,synonymous_variant,missense_variant,upstream_gene_variant | MODERATE,LOW,MODIFIER |
| *Glyma.08G102400* | upstream_gene_variant,intron_variant,3_prime_UTR_variant,downstream_gene_variant | MODIFIER |
| *Glyma.08G102600* | upstream_gene_variant,5_prime_UTR_variant,synonymous_variant,intron_variant,missense_variant,3_prime_UTR_variant,splice_region_variant,downstream_gene_variant | MODERATE,LOW,MODIFIER |
| *Glyma.08G102700* | downstream_gene_variant,3_prime_UTR_variant,intron_variant,synonymous_variant,5_prime_UTR_premature_start_codon_gain_variant,5_prime_UTR_variant,upstream_gene_variant | LOW,MODIFIER |
| *Glyma.08G102900* | downstream_gene_variant,3_prime_UTR_variant,intron_variant,synonymous_variant,upstream_gene_variant | LOW,MODIFIER |
| *Glyma.08G103000* | downstream_gene_variant,stop_lost&splice_region_variant,splice_region_variant&intron_variant,intron_variant,missense_variant,synonymous_variant,splice_donor_variant&intron_variant,upstream_gene_variant | HIGH,MODERATE,LOW,MODIFIER |
| *Glyma.08G103100* | downstream_gene_variant,3_prime_UTR_variant,synonymous_variant,missense_variant,intron_variant,upstream_gene_variant | MODERATE,LOW,MODIFIER |
| *Glyma.08G103200* | upstream_gene_variant,intron_variant,missense_variant,splice_region_variant&intron_variant,synonymous_variant,downstream_gene_variant | MODERATE,LOW,MODIFIER |
| *Glyma.08G103300* | downstream_gene_variant,3_prime_UTR_variant,intron_variant,synonymous_variant,splice_region_variant&intron_variant,upstream_gene_variant | LOW,MODIFIER |
| *Glyma.08G103600* | downstream_gene_variant,3_prime_UTR_variant,upstream_gene_variant | MODIFIER |
| *Glyma.08G104100* | upstream_gene_variant,synonymous_variant,missense_variant,3_prime_UTR_variant,downstream_gene_variant | MODERATE,LOW,MODIFIER |
| *Glyma.08G104300* | downstream_gene_variant,3_prime_UTR_variant,intron_variant,5_prime_UTR_variant,upstream_gene_variant | MODIFIER |
| *Glyma.08G104600* | downstream_gene_variant,3_prime_UTR_variant,synonymous_variant,upstream_gene_variant | LOW,MODIFIER |
| *Glyma.08G105000* | upstream_gene_variant,intron_variant,downstream_gene_variant | MODIFIER |
| *Glyma.08G111700* | downstream_gene_variant,intron_variant,upstream_gene_variant | MODIFIER |
| *Glyma.08G111800* | downstream_gene_variant,3_prime_UTR_variant,intron_variant,5_prime_UTR_premature_start_codon_gain_variant,5_prime_UTR_variant | LOW,MODIFIER |
| *Glyma.08G112000* | upstream_gene_variant,intron_variant,downstream_gene_variant | MODIFIER |
| *Glyma.08G112300* | intron_variant | MODIFIER |
| *Glyma.08G112600* | downstream_gene_variant,3_prime_UTR_variant,upstream_gene_variant | MODIFIER |
| *Glyma.08G113400* | upstream_gene_variant,5_prime_UTR_variant,5_prime_UTR_premature_start_codon_gain_variant,3_prime_UTR_variant,downstream_gene_variant | LOW,MODIFIER |
| *Glyma.08G116900* | upstream_gene_variant,intron_variant,synonymous_variant,missense_variant,missense_variant&splice_region_variant,downstream_gene_variant | MODERATE,LOW,MODIFIER |
| *Glyma.08G117000* | upstream_gene_variant,5_prime_UTR_variant | MODIFIER |
| *Glyma.08G117200* | 5_prime_UTR_variant,intron_variant,downstream_gene_variant | MODIFIER |
| *Glyma.08G118200* | upstream_gene_variant,5_prime_UTR_variant,synonymous_variant,intron_variant,downstream_gene_variant | LOW,MODIFIER |
| *Glyma.08G118600* | downstream_gene_variant,missense_variant,synonymous_variant,5_prime_UTR_variant,upstream_gene_variant | MODERATE,LOW,MODIFIER |
| *Glyma.08G119300* | downstream_gene_variant,3_prime_UTR_variant,intron_variant,splice_region_variant&intron_variant | LOW,MODIFIER |
| *Glyma.08G119700* | upstream_gene_variant,missense_variant,downstream_gene_variant | MODERATE,MODIFIER |
| *Glyma.09G068000* | downstream_gene_variant,3_prime_UTR_variant,intron_variant,5_prime_UTR_variant,5_prime_UTR_premature_start_codon_gain_variant,upstream_gene_variant | LOW,MODIFIER |
| *Glyma.09G068600* | upstream_gene_variant,intron_variant,missense_variant,synonymous_variant,3_prime_UTR_variant,downstream_gene_variant | MODERATE,LOW,MODIFIER |
| *Glyma.09G068700* | downstream_gene_variant,intron_variant,upstream_gene_variant | MODIFIER |
| *Glyma.10G212200* | downstream_gene_variant,intron_variant,upstream_gene_variant | MODIFIER |
| *Glyma.10G212500* | downstream_gene_variant,3_prime_UTR_variant,synonymous_variant,intron_variant,5_prime_UTR_premature_start_codon_gain_variant,5_prime_UTR_variant,upstream_gene_variant | LOW,MODIFIER |
| *Glyma.10G213100* | upstream_gene_variant,intron_variant,downstream_gene_variant | MODIFIER |
| *Glyma.10G213200* | upstream_gene_variant,intron_variant | MODIFIER |
| *Glyma.12G065600* | intron_variant,downstream_gene_variant | MODIFIER |
| *Glyma.12G066200* | downstream_gene_variant,splice_acceptor_variant&intron_variant,intron_variant,missense_variant,upstream_gene_variant | HIGH,MODERATE,MODIFIER |
| *Glyma.12G067700* | upstream_gene_variant,missense_variant,synonymous_variant,intron_variant,3_prime_UTR_variant,downstream_gene_variant | MODERATE,LOW,MODIFIER |
| *Glyma.12G068100* | downstream_gene_variant,3_prime_UTR_variant,intron_variant,synonymous_variant,missense_variant,upstream_gene_variant | MODERATE,LOW,MODIFIER |
| *Glyma.12G068500* | downstream_gene_variant,intron_variant,upstream_gene_variant | MODIFIER |
| *Glyma.15G127400* | upstream_gene_variant,5_prime_UTR_variant,5_prime_UTR_premature_start_codon_gain_variant,intron_variant,3_prime_UTR_variant,downstream_gene_variant | LOW,MODIFIER |
| *Glyma.15G127500* | upstream_gene_variant,5_prime_UTR_variant,synonymous_variant,intron_variant,missense_variant,3_prime_UTR_variant,downstream_gene_variant | MODERATE,LOW,MODIFIER |
| *Glyma.15G127600* | downstream_gene_variant,intron_variant,missense_variant,splice_region_variant&intron_variant,stop_gained,synonymous_variant,upstream_gene_variant | HIGH,MODERATE,LOW,MODIFIER |
| *Glyma.15G127700* | upstream_gene_variant,5_prime_UTR_variant,splice_region_variant&intron_variant,intron_variant,missense_variant,synonymous_variant,3_prime_UTR_variant,downstream_gene_variant | MODERATE,LOW,MODIFIER |
| *Glyma.15G127800* | downstream_gene_variant,3_prime_UTR_variant,intron_variant,splice_region_variant&intron_variant,missense_variant,synonymous_variant,5_prime_UTR_variant,upstream_gene_variant | MODERATE,LOW,MODIFIER |
| *Glyma.15G128000* | upstream_gene_variant,3_prime_UTR_variant,downstream_gene_variant | MODIFIER |
| *Glyma.15G232200* | downstream_gene_variant,3_prime_UTR_variant,missense_variant,5_prime_UTR_premature_start_codon_gain_variant,5_prime_UTR_variant,upstream_gene_variant | MODERATE,LOW,MODIFIER |
| *Glyma.15G232300* | upstream_gene_variant,synonymous_variant,missense_variant,intron_variant,splice_region_variant&synonymous_variant,splice_region_variant&intron_variant,splice_donor_variant&intron_variant,downstream_gene_variant | HIGH,MODERATE,LOW,MODIFIER |
| *Glyma.15G233000* | intron_variant | MODIFIER |
| *Glyma.15G272300* | downstream_gene_variant,synonymous_variant,upstream_gene_variant | LOW,MODIFIER |
| *Glyma.15G273300* | upstream_gene_variant,synonymous_variant,intron_variant,3_prime_UTR_variant,downstream_gene_variant | LOW,MODIFIER |
| *Glyma.15G273600* | upstream_gene_variant,5_prime_UTR_variant,5_prime_UTR_premature_start_codon_gain_variant,intron_variant,synonymous_variant,3_prime_UTR_variant,splice_region_variant,downstream_gene_variant | LOW,MODIFIER |
| *Glyma.15G273900* | downstream_gene_variant,3_prime_UTR_variant,synonymous_variant,intron_variant,missense_variant,upstream_gene_variant | MODERATE,LOW,MODIFIER |
| *Glyma.18G257700* | upstream_gene_variant,intron_variant,synonymous_variant,missense_variant,downstream_gene_variant | MODERATE,LOW,MODIFIER |
| *Glyma.18G257800* | upstream_gene_variant,intron_variant,missense_variant,3_prime_UTR_variant,downstream_gene_variant | MODERATE,MODIFIER |
| *Glyma.18G258000* | upstream_gene_variant,synonymous_variant,missense_variant,3_prime_UTR_variant,downstream_gene_variant | MODERATE,LOW,MODIFIER |
| *Glyma.18G258100* | downstream_gene_variant,3_prime_UTR_variant,intron_variant,synonymous_variant,missense_variant,5_prime_UTR_variant,upstream_gene_variant | MODERATE,LOW,MODIFIER |
| *Glyma.18G258300* | upstream_gene_variant,missense_variant,synonymous_variant,3_prime_UTR_variant,downstream_gene_variant | MODERATE,LOW,MODIFIER |
| *Glyma.18G258500* | upstream_gene_variant,5_prime_UTR_variant,5_prime_UTR_premature_start_codon_gain_variant,synonymous_variant,missense_variant,3_prime_UTR_variant,downstream_gene_variant | MODERATE,LOW,MODIFIER |
| *Glyma.18G259100* | upstream_gene_variant,5_prime_UTR_variant,intron_variant,missense_variant,3_prime_UTR_variant,downstream_gene_variant | MODERATE,MODIFIER |
| *Glyma.18G299400* | downstream_gene_variant,intron_variant,splice_region_variant&intron_variant,upstream_gene_variant,5_prime_UTR_variant | LOW,MODIFIER |
| *Glyma.18G299600* | downstream_gene_variant,splice_region_variant,3_prime_UTR_variant,intron_variant,missense_variant,splice_donor_variant&intron_variant,splice_region_variant&intron_variant,splice_region_variant&synonymous_variant,5_prime_UTR_variant,upstream_gene_variant | HIGH,MODERATE,LOW,MODIFIER |
| *Glyma.18G299700* | upstream_gene_variant,5_prime_UTR_variant,missense_variant,intron_variant,stop_gained,3_prime_UTR_variant,downstream_gene_variant | LOW,MODIFIER |
| *Glyma.18G299900* | upstream_gene_variant,5_prime_UTR_variant,missense_variant,intron_variant,stop_gained,3_prime_UTR_variant,downstream_gene_variant | HIGH,MODERATE,MODIFIER |
| *Glyma.18G300100* | downstream_gene_variant,3_prime_UTR_variant,intron_variant,synonymous_variant,upstream_gene_variant | LOW,MODIFIER |
| *Glyma.18G300200* | upstream_gene_variant,intron_variant,splice_region_variant&intron_variant,synonymous_variant,missense_variant,3_prime_UTR_variant,downstream_gene_variant | MODERATE,LOW,MODIFIER |
| *Glyma.18G300300* | upstream_gene_variant,5_prime_UTR_variant,splice_region_variant,synonymous_variant,missense_variant,intron_variant,3_prime_UTR_variant,downstream_gene_variant | MODERATE,LOW,MODIFIER |
| *Glyma.18G300400* | downstream_gene_variant,3_prime_UTR_variant,missense_variant,intron_variant,synonymous_variant,upstream_gene_variant | MODERATE,LOW,MODIFIER |
| *Glyma.18G300700* | upstream_gene_variant,intron_variant,synonymous_variant,missense_variant,3_prime_UTR_variant,downstream_gene_variant | MODERATE,LOW,MODIFIER |
| *Glyma.20G025900* | downstream_gene_variant,3_prime_UTR_variant,intron_variant,missense_variant,upstream_gene_variant | MODERATE,MODIFIER |
| *Glyma.20G026100* | downstream_gene_variant,intron_variant,missense_variant,synonymous_variant,upstream_gene_variant | MODERATE,LOW,MODIFIER |
| *Glyma.20G026200* | upstream_gene_variant,missense_variant,splice_region_variant&intron_variant,synonymous_variant,intron_variant,downstream_gene_variant | MODERATE,LOW,MODIFIER |
| *Glyma.20G026300* | upstream_gene_variant,intron_variant,synonymous_variant,missense_variant,downstream_gene_variant | MODERATE,LOW,MODIFIER |
| *Glyma.20G026400* | upstream_gene_variant,missense_variant,intron_variant,synonymous_variant,downstream_gene_variant | MODERATE,LOW,MODIFIER |
| *Glyma.20G026700* | downstream_gene_variant,3_prime_UTR_variant,intron_variant,splice_region_variant&intron_variant,missense_variant,synonymous_variant,upstream_gene_variant | MODERATE,LOW,MODIFIER |
| *Glyma.20G026800* | downstream_gene_variant,synonymous_variant,missense_variant,intron_variant,upstream_gene_variant | MODERATE,LOW,MODIFIER |
| *Glyma.20G026900* | upstream_gene_variant,start_lost,missense_variant,synonymous_variant,splice_region_variant&synonymous_variant,intron_variant,stop_gained,missense_variant&splice_region_variant,splice_donor_variant&intron_variant,splice_region_variant&stop_retained_variant,downstream_gene_variant | HIGH,MODERATE,LOW,MODIFIER |
